# Long-Term Survival, Vascular Occlusive Events and Efficacy Biomarkers of First-Line Treatment of CML: A Meta-Analysis

**DOI:** 10.3390/cancers12051242

**Published:** 2020-05-15

**Authors:** Hélène Haguet, Carlos Graux, François Mullier, Jean-Michel Dogné, Jonathan Douxfils

**Affiliations:** 1 Department of Pharmacy, Namur Research Institute for Life Sciences (NARILIS), Namur Thrombosis and Hemostasis Center (NTHC), University of Namur, 5000 Namur, Belgium; jean-michel.dogne@unamur.be (J.-M.D.); jonathan.douxfils@unamur.be (J.D.); 2 Department of Haematology, Namur Research Institute for Life Sciences (NARILIS), Namur Thrombosis and Hemostasis Center (NTHC), CHU UCL Namur, Catholic University of Louvain, 5530 Yvoir, Belgium; carlos.graux@uclouvain.be; 3 Laboratory Hematology, Namur Research Institute for Life Sciences (NARILIS), Namur Thrombosis and Hemostasis Center (NTHC), CHU UCL Namur, Catholic University of Louvain, 5530 Yvoir, Belgium; mullierfrancois@gmail.com; 4 QUALIblood S.A., 5000 Namur, Belgium

**Keywords:** protein kinase inhibitors, overall survival, meta-analysis, leukemia, myelogenous chronic, BCR-ABL positive, arterial occlusive disease

## Abstract

Large randomized clinical trials and prior meta-analyses indicate that second-generation BCR-ABL tyrosine kinase inhibitors (TKIs) improve surrogate biomarkers in patients with chronic myeloid leukemia (CML) without providing survival benefits. The objective is to evaluate the long-term efficacy and the occurrence of vascular occlusion with second-generation BCR-ABL TKIs compared with imatinib in patients with CML. Three scientific databases, a clinical registry and abstracts from congress were searched to identify all randomized controlled trials that compared a second-generation BCR-ABL TKI to imatinib in patients with CML. Outcomes extracted were overall survival, major molecular response and complete cytogenetic response, arterial occlusive events and venous thromboembolism. These data were synthesized by odds ratios using a fixed-effect model. This meta-analysis included 4659 participants from 14 trials. Second-generation BCR-ABL TKIs did not improve overall survival compared with imatinib, even at longer follow-up (OR, 1.17 (95% CI, 0.91–1.52)). They improved surrogate biomarkers at 12 and 24 months but increased the risk of arterial occlusion (OR_PETO_, 2.81 (95% CI, 2.11–3.73)). The long-term benefits of second-generation TKIs are restricted to surrogate outcomes and do not translate into prolonged survival compared to imatinib. Given the long-term use, frontline therapy should be chosen carefully, with special attention to the patients’ quality of life and cardiovascular risks.

## 1. Introduction

### 1.1. Rationale

Treatment of chronic myeloid leukemia (CML) has significantly changed over the last two decades with the development of tyrosine kinase inhibitors (TKIs) targeting BCR-ABL. Today, five BCR-ABL TKIs are approved to treat CML (a sixth BCR-ABL TKI, radotinib, is approved in Korea only). Four of them are indicated for use in newly diagnosed chronic-phase (CP) CML patients [1]. The optimal choice is challenging for physicians. The first-generation TKI, imatinib is a well-known safe and effective drug, whereas second-generation TKIs (i.e., dasatinib, nilotinib or bosutinib) provide faster molecular responses but are considered less safe than imatinib [2,3]. Evidence-based guidelines recommend basing the decision of the frontline therapy on the treatment aim, the treatment cost and the TKI safety profiles [1,4,5]. The use of a second-generation TKI over imatinib is particularly recommended for patients with moderate- or high-risk Sokal scores. Second-generation TKIs are also recommended for young patients because of the higher probability of treatment-free remission with these TKIs [1].

Despite their benefit in molecular and cytogenetic responses, second-generation TKIs have not demonstrated survival benefits over imatinib in clinical trials [6,7], possibly because of the short follow-up. Meta-analyses have been performed to compare the efficacy of second-generation BCR-ABL TKIs with imatinib in patients with CML [8,9,10,11,12]. All concluded that second-generation TKIs provide better surrogate outcomes (i.e., molecular and cytogenetic responses) but no survival benefit [8,10]. However, overall survival analyses were restricted to data at one year and showed high rate of survival. This limits the probability of demonstrating a significant benefit of second-generation TKIs in terms of survival [8]. Since then, additional data from randomized clinical trials (RCTs) with longer follow-up have been published [6,7,13]. The 5-year report of the ENESTnd trial, a large phase 3 trial, revealed that nilotinib induces survival benefits compared with imatinib [14]. In regard to safety, a prior meta-analysis assessed the risk of vascular occlusion of second-generation BCR-ABL TKIs plus the third-generation TKI ponatinib because of a signal during ponatinib development that led to the subsequent discontinuation of the phase 3 trial [15,16]. That study concluded that a greater risk of arterial occlusion was observed compared to the risk with imatinib [17]. Subgroup analyses indicate that an increased risk exists with two of the three second-generation TKIs (dasatinib and nilotinib). The lack of data did not allow firm conclusions about bosutinib, recently approved as a first-line drug for CP-CML [17,18,19]. Since then, the results from an additional phase 3 study have been published on bosutinib. The inclusion of data on long-term follow-up permits a more global approach of the benefit-risk profile of second-generation BCR-ABL TKIs.

### 1.2. Objectives

The study proposed is the first meta-analysis aimed at assessing the long-term overall survival, major molecular response (MMR) and cytogenetic response (CCyR) of second-generation BCR-ABL TKIs compared with imatinib in patients with CML in RCTs. This meta-analysis also compares the occurrence of arterial and venous occlusion with first-line BCR-ABL TKIs in CML patients.

## 2. Results

### 2.1. Eligible Studies and Study Characteristics

**Figure 1:**
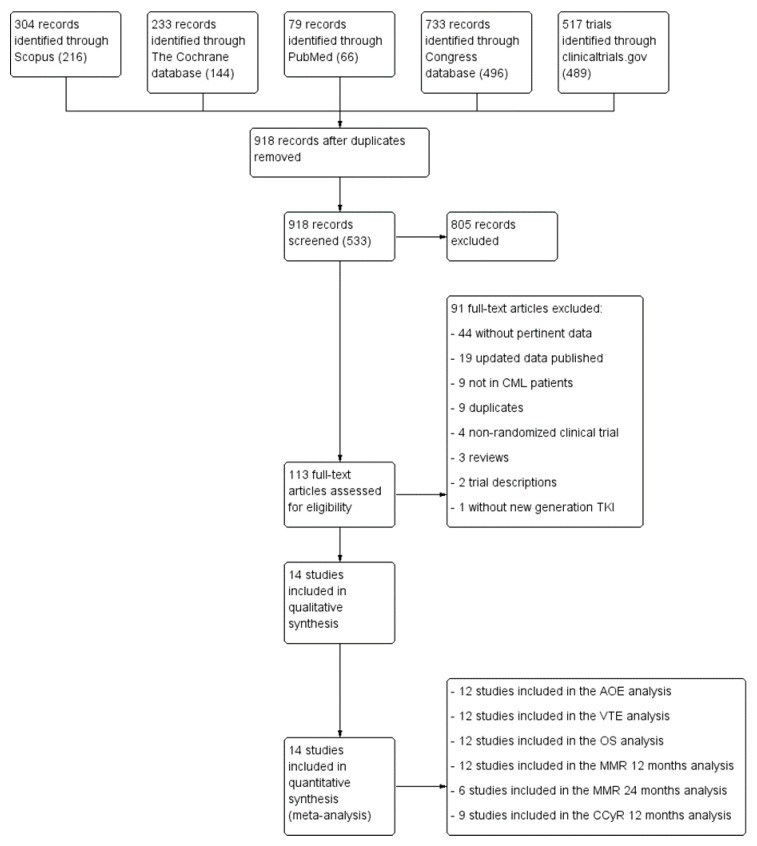
PRISMA flow diagram of the study selection process. Abbreviations: AOE: arterial occlusive event; CCyR: complete cytogenetic response; MMR: major molecular response; OS: overall survival; TKI: tyrosine kinase inhibitor; VTE: venous thromboembolism.

The literature search yielded 918 records. After title and abstract screening, 113 full texts were considered for further investigation. The full-text screening excluded 93 articles. Major exclusion reasons were the lack of pertinent data, outdated data or an inappropriate study design. Finally, we identified 14 clinical trials reported in 20 abstracts and articles that met the inclusion criteria, involving a total of 4659 participants [6,7,13,20,21,22,23,24,25,26,27,28,29,30,31,32,33,34,35,36,37,38]. Figure 1 shows the flow of studies in the systematic review process and Table S1 lists the key characteristics of the 14 studies. All included studies are RCTs.

The risk of bias for each of the 14 included studies is shown in Figure S1.

### 2.2. Overall Survival

**Figure 2:**
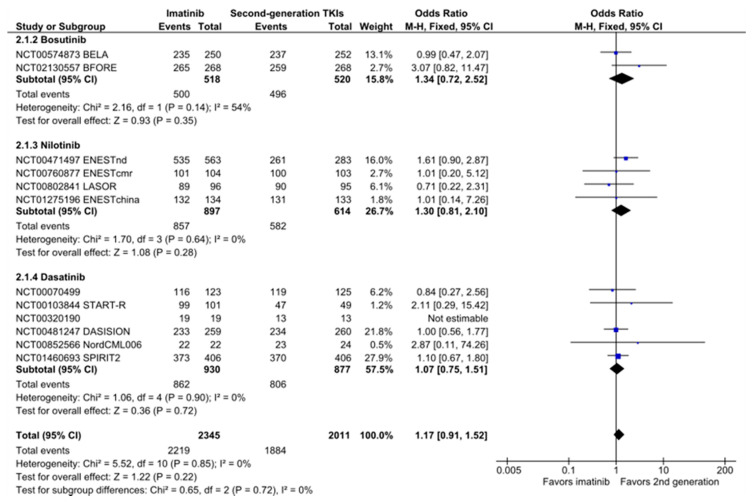
Forest plot of overall survival in patients with chronic myeloid leukemia (CML) treated with second-generation TKIs versus imatinib.

Of the 14 included studies, 12 reported overall survival data. There was no significant difference between second-generation TKIs and imatinib in the outcome of overall survival (OR, 1.17 (95% CI: 0.91–1.52); Figure 2). This result was consistent between TKIs (subgroup difference, I^2^ = 0%; Figure 2) and between nilotinib dose regimens (300 mg BID: OR, 1.21 (95% CI: 0.66 to 2.24); 400 mg BID: OR, 1.53 (95% CI: 0.86–2.74); Figure S2). The anticipated absolute effect of overall survival was 0.9% superior (95% CI: −0.6–2.1) with second-generation TKIs over imatinib (survival rate of 93.7%, Table 1). The omission of clinical trials performed in previously treated CML patients did not significantly change the results (OR, 1.20 (95% CI: 0.92 to 1.57); Figure S3). Only two studies reported overall survival in clinical trials comparing high-dose imatinib to second-generation TKIs. Their analysis demonstrated no survival benefit of high-dose imatinib compared to second-generation TKIs (Figure S3 and Table S2).

### 2.3. Efficacy

The MMR rate at 12 and 24 months and the CCyR rate at 12 months were reported in 12, 6 and 9 studies respectively. Of these three efficacy outcomes, all favored second-generation TKIs (MMR 12 months: OR, 2.01 (95% CI: 1.77–2.30); MMR 24 months: OR, 1.40 (95% CI: 1.17–1.67)); CCyR 12 months: OR, 1.50 (95% CI: 1.31–1.72); Figure S4), even compared with high-dose imatinib (Figure S3). These results were consistent between studies for MMR at 12 and 24 months (respectively, I^2^ = 20% and 0%), but the I^2^ statistic indicated moderate heterogeneity for CCyR at 12 months (I^2^ = 48%). This inconsistency was mainly due to the heterogeneity between nilotinib (I^2^ = 81%) and bosutinib trials (I^2^ = 64%). Differences between these study designs were related to TKI dose and population (i.e., patients previously treated vs. treatment-naïve patients; Table S1). The stratification by nilotinib dose regimen demonstrated similar results between 300 and 400 mg BID (Figure S2), and the omission of clinical trials performed in previously treated CML patients did not significantly change the results (Figure S3).

### 2.4. Vascular Occlusion

**Figure 3:**
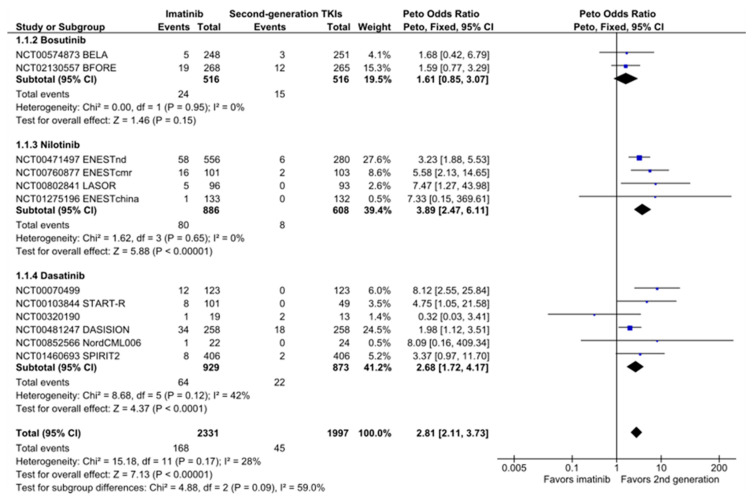
Forest plot of arterial occlusive events in patients with CML treated with second-generation TKIs versus imatinib.

The risk of AOEs was increased with second-generation TKIs compared with imatinib (OR_PETO_, 2.81 (95% CI: 2.11–3.73); Figure 3) and high-dose imatinib (Figure S3). With imatinib, 23 out of 1000 patients developed an arterial occlusion, whereas this meta-analysis estimated that this rate increased to 61 out of 1000 patients with second-generation TKIs (95% CI: 46–79, Table 2). There was a trend toward an increased risk of venous thromboembolism (VTE) (OR_PETO_, 1.74 (95% CI: 0.82–3.66); Figure S4).

The risk of AOEs was consistently higher for second-generation TKIs and the difference was significant for nilotinib (OR, 3.89 (95% CI: 2.47–6.11)) and dasatinib (OR, 2.68 (95% CI: 1.72–4.17)). Only bosutinib was not associated with a significantly increased risk of AOEs compared with imatinib (OR, 1.61 (95% CI: 0.85–3.07)). However, this result became significant when simulating recruitment similar to that with dasatinib and nilotinib (i.e., repeating the analysis until the same number of patients was obtained). Arterial occlusive events (AOEs) occurred more frequently with nilotinib 400 mg BID than with nilotinib 300 mg BID (300 mg BID: 3.32 (95% CI: 1.56–7.09); 400 mg BID: 5.19 (95% CI: 3.14–8.57); Figure S2).

### 2.5. Risk of Bias

The analysis of the risk of bias is reported in the Figure S1. Four studies of the 12 included in the AOE analysis were considered to have high concern. Indeed, because of the discovery of the risk of vascular occlusion with another BCR-ABL TKI (i.e., ponatinib) before these four studies were conducted, the reporting and the subjectivity of similar events (e.g., chest pain, phlebitis) with the investigated treatments might have been affected. All of the 14 included trials were at moderate risk of bias because of the high rate of treatment discontinuation or switching and the possibility of modifying the treatment dose. Additional risks of potential bias were the lack of details of the randomization process, the selection of the reported outcome and the open-label design. Funnel plots and Egger’s tests indicated no evidence of publication bias (Figure S5).

## 3. Discussion

### 3.1. Should Second-Generation TKIs Be Favored over Imatinib for Treatment-Naïve CP-CML Patients?

#### 3.1.1. Are Second-Generation TKIs More Efficient Than Imatinib to Treat CP-CML?

This is the first meta-analysis of second-generation BCR-ABL TKIs that provides long-term estimates on efficacy and safety compared to imatinib. The major finding of this meta-analysis of 14 RCTs including 4659 CML patients was that second-generation BCR-ABL TKIs did not significantly improve short- or long-term overall survival compared with imatinib. Except for nilotinib, for which survival superiority was suggested in the ENESTnd trial [14], this absence of the benefit of second-generation TKI in terms of survival is in accordance with the RCT results [6,7]. Unsurprisingly, and in line with large clinical trials, surrogate outcomes such as the rates of MMR and CCyR at 12 and 24 months were improved with second-generation BCR-ABL TKIs compared to imatinib.

Based on the higher rate of surrogate outcomes with second-generation TKIs, numerous recent studies have concluded that second-generation TKIs are superior for frontline treatment of CML, challenging the position of imatinib [2,9,10,12]. Surrogate outcomes have been adopted as predictive of overall survival at the time CML patients were treated with interferon-α because of the strong correlation between cytogenetic response and survival. They have then been used for TKI marketing authorization because patient-relevant outcomes (e.g., overall survival and progression-free survival) are time dependent. However, BCR-ABL TKIs and interferon-α differ in their mechanisms of action, but the validity of the surrogate outcomes to predict patient survival with TKIs has not been questioned. Different molecular responses (early molecular response and MR 4.5) have been proposed as surrogate markers for overall survival but have failed to predict patient survival [39]. To our knowledge, it has not been demonstrated that a potential TKI effect on survival is explained by molecular response. These biomarkers may not fully capture the action TKIs have on the overall survival. Indeed, the lack of selectivity of BCR-ABL TKIs is responsible for numerous adverse events that may affect patient survival (e.g., ischemic stroke or myocardial infarction) [40]. A long-term study of patients with CML treated by second- or third- generation TKIs demonstrates that approximately a third of death were related to cardiovascular diseases [41]. This potentially explains why, even with long follow-up, improvement in surrogate outcomes with second-generation TKIs does not result in better overall survival. Consequently, surrogate outcomes should not be interpreted alone. The long-term benefits, treatment safety and patients’ quality of life (QoL) should be preferred and seem more relevant because of the long life expectancy of CP-CML patients, close to that of the general population [42].

#### 3.1.2. Do All Second-Generation TKIs Favor Arterial Occlusion?

As imatinib and second-generation TKIs confer similar survival probability, their vascular safety is important to consider avoiding premature non-CML-related deaths or vascular-related disabilities. Our meta-analysis confirms that the risk of AOEs is higher with second-generation TKIs than with imatinib. Of 1000 CP-CML patients, 38 more patients (95% CI, 24–57) will develop an arterial occlusive disease with second-generation TKIs compared with imatinib. In accordance with real-life studies, the risk of arterial occlusion has reportedly been higher with nilotinib, dasatinib and ponatinib. Bosutinib has the safer vascular profile and is currently not associated with a significant increase of the risk of vascular occlusion [43,44]. However, the size of the population of the bosutinib analysis is limited compared to the number of patients in the dasatinib and nilotinib subgroups. This lack of statistical evidence should therefore not be seen as evidence of an absence of a true risk. Further investigations are needed to improve the power of the analysis. In addition, data with bosutinib were obtained from two trials sponsored by the marketing authorization holder. Assessment of the AOE analysis indicated that higher ORs were reported in institutional or academic trials (i.e., NCT00070499 and NordCML006) than in sponsored industrial studies, raising some concerns about the risk of bias.

#### 3.1.3. Imatinib and Second-Generation TKIs Have Limited Impact on Patients’ QoL

The potential long-term duration of BCR-ABL TKI treatment led patients and physicians to pay more attention to treatment long-term risks and the impact on patients’ QoL. However, the latter has rarely been studied in clinical trials. The few such studies report similar QoL with imatinib as in the general population among older patients (older than 59 years) [45]. However, younger patients are more affected and report limitations in work and daily activities compared to the age-matched general population (i.e., without cancer) [45]. With second-generation TKIs, quality-of-life sub analyses have been performed in four large RCTs (i.e., BFORE, ENESTnd, ENESTchina and SPIRIT2) [46,47,48,49,50,51]. All these analyses reported no differences in QoL between the second-generation TKIs and imatinib [46,47,48,49,50,51].

#### 3.1.4. Recommendations for Frontline Treatment of CP-CML

Imatinib and second-generation BCR-ABL TKIs are all highly effective in treating CP-CML and provide survival close to that of the age-matched population. Imatinib has demonstrated reassuring long-term safety and efficacy [52]. At 10 years, the overall survival rate was estimated to be 83.3%, and fewer than 10% of patients developed serious adverse events [52]. Second-generation TKIs increase the rate of molecular and cytogenetic responses but do not provide benefits in patient survival or QoL compared with imatinib.

The induction of faster and deeper molecular responses with second-generation TKIs compared to imatinib in treatment-naïve patients has generated interest over the last few years for these drugs because of the increasing number of candidates for TKI discontinuation (i.e., a higher number of patients who achieved rapid molecular remission) [53]. However, in retrospect, treatment discontinuation was less successful than announced, with a higher number of patients relapsing after TKI cessation than expected. Recent studies indicate that TKI discontinuation was successful in 20% of patients (i.e., almost 40% of CML patients are eligible for treatment discontinuation, with a success rate of 50%), and criteria for trying treatment cessation are more constraining [54]. In addition, patients should be treated longer (i.e., optimally more than 8 years) with a TKI before trying treatment cessation [54].

Due to the safety profile of second-generation TKIs, particularly the vascular issue damage from these treatments (with the exception of bosutinib, which requires further investigation), the frontline treatment choice should not focus solely on the objective of treatment-free remission. Arterial occlusion occurred more frequently in patients with prior cardiovascular risk factors. Therefore, an estimation of 10-year arteriosclerotic cardiovascular disease risk, as recommended by the ACC/AHA, should be applied to CML patients before making a treatment decision [55].

The treatment choice should then be made individually by carefully weighing the benefits and the risks of each TKI. These results support the current NCCN guidelines, particularly to limit the use of second-generation TKIs as frontline drugs for young patients. Finally, the treatment cost should be taken into account. A recent study demonstrates that the one-year health care expenditures were significantly higher among patients treated with second-generation BCR-ABL TKIs as first-line, compared with those treated with imatinib [56].

#### 3.1.5. Is the BCR-ABL TKI Dose Optimal?

Modification of the TKI dose regimen has already been proposed as a potential measure to minimize the risk of AOEs, but data were insufficient to provide formal recommendations [8]. The results of our meta-analysis suggest that the efficacy of nilotinib is similar independent of the dose. However, nilotinib 400 mg BID was associated with a higher incidence of AOEs. Therefore, the use of 400 mg BID nilotinib is questionable. Dasatinib has been tested in a RCT only at 100 mg QD in newly diagnosed CP-CML patients. Future studies should assess the benefits of reduced doses of nilotinib and dasatinib to minimize the risk of arterial occlusion while preserving efficacy. For bosutinib, the comparison between the BELA and the BFORE trial, in which participants were treated with 500 mg and 400 mg QD, respectively, indicates a similar OR for efficacy outcomes and AOE occurrence. A dose reduction to 300 mg QD allowed a reduction of adverse events and better tolerability. However, its effect on efficacy and arterial occlusion has not been studied [57].

### 3.2. Measures to Minimize the Vascular Risk with Second-Generation TKIs

Several measures have already been recommended in the literature to minimize the risk of arterial occlusion [17,58]. These recommendations aim to minimize cardiovascular risk by acting on common cardiovascular risk factors. Prevention treatment is only recommended for patients with diagnosed cardiovascular disease or diabetes. In the European Summary of Product Characteristics (EU-SMPC), recommendations to prevent arterial occlusion are only provided for nilotinib [59,60,61]. The SMPC of nilotinib does not suggest additional measures that evaluate cardiovascular risk or the management or monitoring of common cardiovascular risk factors. Recent in vitro and in vivo studies suggest that nilotinib and ponatinib may induce arterial occlusion through coronary artery vasospasm by inhibiting the RAS/RAF/MEK pathway and increasing Ca^2+^ release by activating the calcium channel [62,63]. The use of diltiazem, a calcium channel blocker, to prevent vascular occlusion is an interesting possibility that should be further investigated in clinical settings with nilotinib and ponatinib [63].

### 3.3. Strengths and Limitations

A major limitation of this meta-analysis is the use of aggregate data from the studies because of the lack of access to individual data. This precludes additional calculations such as overall survival analyses based on patients’ Sokal score and age and the integration of patients’ comorbidities in the analysis of AOEs. In addition, access to individual data would provide the advantage that statistical analyses could be standardized between but also within studies, and allow the computation of the number of patients eligible for treatment discontinuation. Progression-free survival was not analyzed in our meta-analysis as its definition differs between studies [64]. Another limitation was the selection of the ITT population as data source, as this population does not take into account the proportion of patients who switched to another TKI.

A major strength of this meta-analysis is the integration of both published and unpublished data that limit the risk of publication bias. As much as possible, we confront the data from the different sources and use a conservative approach to select the data that have been included in the quantitative analysis. Another strength of our analysis is that we assessed the rates of molecular and cytogenetic responses at specific time points (e.g., MMR at 12 months). Most clinical trials have investigated these surrogate outcomes over a period of time, also called the cumulative response, and the reporting of these data is often unclear (i.e., no specification of the type of analysis). Due to the rapid variation of the molecular response, the cumulative incidence is frequently higher than the response rate at a specific time point. Loss of molecular or cytogenetic responses can be the consequence of lack of compliance, treatment intolerance and drug resistance, three factors that are revealed by the analysis of responses at specific time points rather than cumulative response.

## 4. Materials and Methods

### 4.1. Search Methods

This meta-analysis was performed in accordance with the protocol for meta-analyses published in 2015 [8] and complied with the PROSPERO protocol 2014:CRD42014014147. Three scientific databases (PubMed (from 1966), Scopus (from 1995) and CENTRAL (Cochrane Central Register of Controlled Trials; from 1996)), a clinical trial registry (clinicaltrials.gov) and abstracts from the last 3 years of 3 meetings (i.e., ASCO, ESMO and ASH congresses) were searched up to 14 January 2019 using the search strategy reported in Method S1. Only English publications were considered. This meta-analysis follows the PRISMA statement (Table S3).

### 4.2. Data Collection

#### 4.2.1. Study Selection

Two researchers (HH and JD) independently screened all titles and abstracts identified from the literature search to determine potentially eligible studies. We included RCTs that compared a second-generation TKI approved for first-line CML treatment with imatinib in patients with CML. The full texts of these potentially relevant articles were then assessed independently by the same researchers (HH and JD) based on predefined inclusion and exclusion criteria. Disagreements were resolved through discussion with a third author (JMD).

#### 4.2.2. Data Extraction

The data extraction was performed for each study independently by 2 researchers (HH and JD) using a standard data extraction form. The outcomes extracted were overall survival, MMR and CCyR at predefined time points (12 months, 24 months, 36 months, 48 months and 60 months, when available), as well as AOEs and VTEs. Method S2 presents the list of terms considered as AOEs and VTEs. Reviewers took special care to extract responses at a specific time point rather than the cumulative response (i.e., rate of response achieved by the time point, regardless of whether they lost the response/discontinued or not). The number of patients alive was also preferred over the estimated overall survival (Kaplan–Meier analysis), and the overall survival with the longest study follow-up was included.

When discrepancies occurred between sources a conservative approach was adopted. For AOE and VTE analyses, data were extracted from the adverse event report rather than from secondary analyses performed by the study sponsor (i.e., vascular safety analyses performed as secondary outcomes) when possible, to limit the risk of bias.

### 4.3. Data Analysis

#### 4.3.1. Data Synthesis

We conducted meta-analyses of dichotomous variables with the fixed-effect model (FEM) by computing odds ratios (ORs) and 95% confidence intervals (CIs). For the AOE and VTE analyses, ORs were computed using the Peto method because the rate of events was low [65]. Statistical analyses were performed using Review Manager (RevMan) version 5.3 (Copenhagen, 2014, https://training.cochrane.org/online-learning/core-software-cochrane-reviews/revman/revman-5-download). In addition to ORs, anticipated absolute effects were computed using a GRADE profiler (GRADEpro GDT, McMaster University, 2015). This measure was expressed as a risk difference with its 95% CI and is based on the baseline risk (i.e., the risk in the comparison group) and the relative effect of the intervention.

#### 4.3.2. Subgroup Analysis and Investigation of Heterogeneity

Stratification per treatment was performed, as well as per TKI dose when data were available. Heterogeneity among trials was quantified by the I^2^ statistic, with an I^2^ value < 25% reflecting mild heterogeneity, 25%–50% reflecting moderate heterogeneity and > 50% reflecting severe heterogeneity.

#### 4.3.3. Sensitivity Analysis

Sensitivity analyses were performed as a repeat of the meta-analysis by substituting previously treated patients in order to explore their impact on MMR and CCyR. Sensitivity analyses were also performed by subtracting trials with high-dose imatinib (i.e., greater than 400 mg per day).

#### 4.3.4. Assessment of Risk of Bias in Included Studies

The risk of bias was assessed using the Cochrane Collaboration’s risk of bias 2 (RoB 2) tool for RCT [66]. To assess the risk of reporting bias, we carried out an assessment of publication bias using funnel plots. Funnel plot asymmetry was evaluated using Egger’s regression tests [67].

## 5. Conclusions

Since the introduction of BCR-ABL TKIs, patients’ and physicians’ concerns have shifted because of their high efficacy, and more attention has been paid to treatment toxicity and patients’ QoL. Even with long follow-up, there is no direct evidence of a survival benefit associated with second-generation BCR-ABL TKIs. Given the long-term use of BCR-ABL TKI treatment, the therapeutic option should be selected individually and based on multiple factors, including cardiovascular safety and QoL. Future research should focus on these two last points. To date, the cardiovascular safety of bosutinib is reassuring. In light of the present results, it is anticipated that an in-depth reanalysis of the clinical trials by the marketing authorization holders together with the regulatory bodies would be needed to update the product information with the most recent data analysis. This would provide health care professionals with updated recommendations on the benefits and risks of these medicines.

## Figures and Tables

**Table 1:** Overall survival in patients with CML receiving second-generation BCR-ABL TKIs. (The bold is a summary of the other rows.)

BCR-ABL TKI No. of Patients (No. of Studies)	Odds Ratio (95% CI)	Anticipated Absolute Effects (95% CI)		
		Risk with Imatinib	Risk with 2nd-Generation TKI	Risk Difference with 2nd-Generation TKI
**All 2nd Generation TKI** **No. of Patients: 4356** (**12 Studies**)	**1.17** (**0.91 to 1.52**)	**93.7%**	**94.6%** (**93.1 to 95.8**)	**+0.9%** (**−0.6 to +2.1**)
Bosutinib No. of Patients: 1038 (2 Studies)	1.34 (0.72 to 2.52)	95.4%	96.5% (93.7 to 98.1)	+1.1% (−1.7 to +2.7)
Nilotinib No. of Patients: 1511 (4 Studies)	1.30 (0.81 to 2.10)	94.8%	95.9% (93.6 to 97.4)	+1.2% (−1.1 to +2.7)
Dasatinib No. of Patients: 1807 (6 Studies)	1.07 (0.75 to 1.51)	91.9%	92.4% (89.5 to 94.5)	+0.5% (−2.4 to +2.6)

**Table 2:** Arterial occlusive events in patients with CML receiving second-generation BCR-ABL TKIs. (The bold is a summary of the other rows.)

BCR-ABL TKI No. of Patients (Studies)	Odds Ratio (95% CI)	Anticipated Absolute Effects (95% CI)		
		Risk with Imatinib	Risk with 2nd Generation TKI	Risk Difference with 2nd Generation TKI
**All 2nd Generation TKI** **No. of Patients: 4328** **(12 RCTs)**	**OR 2.81** **(2.11 to 3.73)**	**2.3%**	**6.1%** **(4.6 to 7.9)**	**+3.8%** **(+2.4 to +5.7)**
BOSUTINIB No. of patients: 1032 (2 RCTs)	OR 1.61 (0.85 to 3.07)	2.9%	4.6% (2.5 to 8.4)	+1.7% (−0.4 to +5.5)
Nilotinib No. of Patients: 1494 (4 RCTs)	OR 3.89 (2.47 to 6.11)	1.3%	4.9% (3.2 to 7.5)	+3.6% (+1.9 to +6.2)
Dasatinib No. of Patients: 1802 (6 RCTs)	OR 2.68 (1.72 to 4.17)	2.5%	6.5% (4.3 to 9.7)	+4.0% (+1.7 to +7.2)

## Supplementary Materials

The following are available online at https://www.mdpi.com/2072-6694/12/5/1242/s1, Method S1: Search strategy, Method S2: list of terms considered as “arterial occlusive events” and “venous thromboembolisms”, Figure S1: Risk of bias summary, Figure S2: Sub-analyses by TKI dose, Figure S3: Sensitivity analysis, Figure S4: Forest plots of MMR, CCyR and venous thromboembolism in patients with CML treated with second generation TKIs compared with imatinib, Figure S5: Publication bias assessment, Table S1: Main characteristics of the 14 included clinical trials, Table S2: Detailed statistics in Figure S3, Table S3: PRISMA checklist.
