# Supplementary material for: Long-Term Survival, Vascular Occlusive Events and Efficacy Biomarkers of First-Line Treatment of CML: A Meta-Analysis
Source: Cancers (Basel). 2020 May 15;12(5):1242. doi: 10.3390/cancers12051242 (PMC7281573; doi:10.3390/cancers12051242)
Supplement: Supplementary file 1 [file cancers-12-01242-s001.pdf]

Supplementary Materials

Long-Term Survival, Vascular Occlusive Events and Efficacy Biomarkers of First-Line Treatment of CML: A Meta-Analysis

Hélène Haguet, Carlos Graux, François Mullier, Jean-Michel Dogné and Jonathan Douxfils

Method S1: Search strategy

A) The Cochrane Library (From November, 2016 to January 14th, 2019)

Bosutinib

1. bosutinib OR SKI-606 OR SKI606 (84 articles)
2. "randomized controlled trial" or "randomized trial" or "randomized clinical trial" or "randomised controlled trial" or "randomised trial" or "randomised clinical trial" (684885 articles)
3. #1 AND #2
   "Search limits" → Content type: "Trials" + Cochrane Library publication date: between "Nov

   2016" (22 articles)

Ponatinib

1. ponatinib or AP24534 (63 articles)
2. "randomized controlled trial" or "randomized trial" or "randomized clinical trial" or "randomised controlled trial" or "randomised trial" or "randomised clinical trial" (684885 articles)
3. #1 AND #2
   "Search limits" → Content type: "Trials" + Cochrane Library publication date: between "Nov

   2016" (11 articles)

Nilotinib

1. nilotinib or AMN107 (301 articles)
2. "randomized controlled trial" or "randomized trial" or "randomized clinical trial" or "randomised controlled trial" or "randomised trial" or "randomised clinical trial" (684885 articles)
3. #1 AND #2
   "Search limits" → Content type: "Trials" + Cochrane Library publication date: between "Nov

   2016" (51 articles)

Dasatinib

1. dasatinib or BMS-354825 or BMS354825 (332 articles)

2. "randomized controlled trial" or "randomized trial" or "randomized clinical trial" or "randomised controlled trial" or "randomised trial" or "randomised clinical trial" (684885 articles)
3. #1 AND #2
   "Search limits" → Content type: "Trials" + Cochrane Library publication date: between "Nov

   2016" (53 articles)

Imatinib

1. imatinib or imatinib mesylate or STI-571 or STI571 (1097 articles)
2. "randomized controlled trial" or "randomized trial" or "randomized clinical trial" or "randomised controlled trial" or "randomised trial" or "randomised clinical trial" (684885 articles)
3. #1 AND #2
   "Search limits" → Content type: "Trials" + Cochrane Library publication date: between "Nov

   2016" (131 articles)

Radotinib

1. Radotinib or IY5511 or IY-5511 (9 articles)
2. "randomized controlled trial" or "randomized trial" or "randomized clinical trial" or "randomised controlled trial" or "randomised trial" or "randomised clinical trial" (703781 articles)
3. #1 AND #2
   "Search limits" → Content type: "Trials" + Cochrane Library publication date: to Jan 2019 (5

   articles)

Total

Combination of the searches with « OR » (179 articles)

B) PubMed (From November, 8th 2016 to January, 14th 2019)

Dasatinib

1. Search **((dasatinib[Title]) OR BMS-354825[Title]) OR BMS354825[Title]**
   (1143 Articles)

2. Search **(((((randomized controlled trial) OR randomized trial) OR randomized clinical trial) OR randomised controlled trial) OR randomised trial) OR randomised clinical trial**
   (684679 articles)

3. #1 AND #2 AND ("2016/11/08"[Date - Publication]: "3000"[Date - Publication])
   (7 articles)

Nilotinib

1. Search **(nilotinib[Title]) OR AMN107[Title]**

(711 Articles)

2. Search **(((((randomized controlled trial) OR randomized trial) OR randomized clinical trial) OR randomised controlled trial) OR randomised trial) OR randomised clinical trial**
(684679 articles)

3. #1 AND #2 AND ("2016/11/08"[Date - Publication]: "3000"[Date - Publication])
(6 articles)

Bosutinib

1. Search **((bosutinib[Title]) OR SKI-606[Title]) OR SKI606[Title]**
(136 Articles)

2. Search **(((((randomized controlled trial) OR randomized trial) OR randomized clinical trial) OR randomised controlled trial) OR randomised trial) OR randomised clinical trial**
(684679 articles)

3. #1 AND #2 AND ("2016/11/08"[Date - Publication]: "3000"[Date - Publication])
(5 articles)

Ponatinib

1. Search **(ponatinib[Title]) OR AP24534[Title]**
(210 Articles)

2. Search **(((((randomized controlled trial) OR randomized trial) OR randomized clinical trial) OR randomised controlled trial) OR randomised trial) OR randomised clinical trial**
(684679 articles)

3. #1 AND #2 AND ("2016/11/08"[Date - Publication]: "3000"[Date - Publication])
(2 articles)

Imatinib

1. Search **(((imatinib[Title]) OR imatinib mesylate[Title]) OR STI-571[Title]) OR STI571[Title]**
(6285 Articles)

2. Search **(((((randomized controlled trial) OR randomized trial) OR randomized clinical trial) OR randomised controlled trial) OR randomised trial) OR randomised clinical trial**
(684679 articles)

3. #1 AND #2 AND ("2016/11/08"[Date - Publication]: "3000"[Date - Publication])
(21 articles)

Radotinib

1. Search **((radotinib[Title]) OR IY5511[Title]) OR IY-5511[Title]** (19 articles)

2. Search **(((((randomized controlled trial) OR randomized trial) OR randomized clinical trial) OR randomised controlled trial) OR randomised trial) OR randomised clinical trial** (688374 articles)

3. #1 AND #2 (1 article)

Total

Combination with "OR" (exemple: ((((#20) OR #18) OR #16) OR #14) OR #8)
(33 articles)

C) Scopus (From 2016 to January 14th, 2019)

Bosutinib

(TITLE (bosutinib) OR TITLE (ski-606) OR TITLE (SKI606)) AND (ALL ("randomized controlled trial") OR ALL ("randomized trial") OR ALL ("randomized clinical trial") OR ALL ("randomised controlled trial") OR ALL("randomised trial") OR ALL ("randomised clinical trial"))
Articles published between 2016 and 2019 (8 articles)

Ponatinib

(TITLE (ponatinib) OR TITLE (AP24534)) AND (ALL ("randomized controlled trial") OR ALL ("randomized trial") OR ALL ("randomized clinical trial") OR ALL ("randomised controlled trial") OR ALL("randomised trial") OR ALL ("randomised clinical trial"))
Articles published between 2016 and 2019 (14 articles)

Nilotinib

(TITLE (nilotinib) OR TITLE (AMN107)) AND (ALL ("randomized controlled trial") OR ALL ("randomized trial") OR ALL ("randomized clinical trial") OR ALL ("randomised controlled trial") OR ALL("randomised trial") OR ALL ("randomised clinical trial"))
Articles published between 2016 and 2019 (20 articles)

Dasatinib

(TITLE (dasatinib) OR TITLE (BMS-354825) OR TITLE (BMS354825)) AND (ALL ("randomized controlled trial") OR ALL ("randomized trial") OR ALL ("randomized clinical trial") OR ALL ("randomised controlled trial") OR ALL("randomised trial") OR ALL ("randomised clinical trial"))
Articles published between 2016 and 2019 (36 articles)

Imatinib

(TITLE (imatinib) OR TITLE (imatinib mesylate) OR TITLE (STI-571) OR TITLE (STI571)) AND (ALL ("randomized controlled trial") OR ALL ("randomized trial") OR ALL ("randomized clinical trial") OR ALL ("randomised controlled trial") OR ALL("randomised trial") OR ALL ("randomised clinical trial"))
Articles published between 2016 and 2019 (169 articles)

Radotinib

(TITLE (radotinib) OR TITLE (iy5511) OR TITLE (iy-5511)) AND (ALL ("randomized controlled trial") OR ALL ("randomized trial") OR ALL ("randomized clinical trial") OR ALL ("randomised controlled trial") OR ALL ("randomised trial") OR ALL ("randomised clinical trial")) (3 articles)

Total

(TITLE (imatinib) OR TITLE (imatinib mesylate) OR TITLE (STI-571) OR TITLE (STI571) OR TITLE (dasatinib) OR TITLE (BMS-354825) OR TITLE (BMS354825) OR TITLE (nilotinib) OR TITLE (AMN107) OR TITLE (ponatinib) OR TITLE (AP24534) OR TITLE (bosutinib) OR TITLE (ski-606) OR TITLE (SKI606)) AND (ALL ("randomized controlled trial") OR ALL ("randomized trial") OR ALL ("randomized clinical trial") OR ALL ("randomised controlled trial") OR ALL("randomised trial") OR ALL ("randomised clinical trial"))

Articles published between 2016 and 2019 (230 articles)

D) Meeting abstracts

ASCO

- ASCO annual meeting 2017

Title:"imatinib" OR Title:"dasatinib" OR Title:"nilotinib" OR Title:"bosutinib" OR Title:"ponatinib" OR Title:"radotinib" (22 abstracts)

- ASCO annual meeting 2018

Title:"imatinib" OR Title:"dasatinib" OR Title:"nilotinib" OR Title:"bosutinib" OR Title:"ponatinib" (21 abstracts)

ESMO

- ESMO 2017

Advanced search → « presentation title »: « imatinib dasatinib nilotinib bosutinib ponatinib radotinib » (9 abstracts)

- ESMO 2018

Advanced search → « presentation title »: « imatinib dasatinib nilotinib bosutinib ponatinib radotinib » (4 abstracts)

ASH

- ASH 2016

Imatinib OR ponatinib OR nilotinib OR bosutinib OR dasatinib OR radotinib (96 abstracts)

- ASH 2017

Imatinib OR ponatinib OR nilotinib OR bosutinib OR dasatinib OR radotinib (71 abstracts)

- ASH 2018

Imatinib OR ponatinib OR nilotinib OR bosutinib OR dasatinib OR radotinib (86 abstracts)

Method S2: List of terms considered as "arterial occlusive events" and "venous thromboembolism".

Arterial occlusive events	Venous thromboembolism
acute coronary syndrome	deep vein thrombosis
acute myocardial infarction	intermittent claudication
angina pectoris	phlebitis
angina unstable	retinal vein occlusion
aortic stenosis	retinal vein thrombosis
arterial occlusive disease	thrombophlebitis
arterial stenosis limb	varicose vein
arteriosclerosis	venous thrombosis limb
arteriosclerosis coronary artery	vasculitis
atrial thrombosis	thrombocytopenic purpura
basilar artery stenosis	pulmonary embolism
cardiogenic shock	
cardio-respiratory arrest	
cerebral infarction	
cerebrovascular accident	
cerebrovascular disorder	
chest discomfort	
chest pain	
coronary artery disease	
coronary artery occlusion	
coronary artery stenosis	
electrocardiogram ST-T segment abnormal	
femoral arterial stenosis	
iliac artery occlusion	
iliac artery stenosis	
ischaemic stroke	
myocardial ischaemia	
peripheral artery occlusive disease	
peripheral artery thrombosis	
sudden death	
transient ischemic attack	
vena cava thrombosis	
cerebrovascular ischemia	
cardiac ischemia/infarction	
cardiac arrest	
myocardial infarction	

(A). Overall survival

Study ID	Experimental	Weight	Randomization process	Deviations from intended interven	Missing outcome data	Measurement of the outcome	Selection of the reported re sult	Overall
NCT00574875	Bosutinib	12,8	+	?	+	+	+	!
NCT02130557	Bosutinib	2,6	+	?	+	+	+	!
NCT00471497	Nilotinib	15,6	+	?	+	+	+	!
NCT00760877	Nilotinib	2,6	?	?	+	+	+	!
NCT01275196	Nilotinib	1,8	?	?	+	+	+	!
NCT00802841	Nilotinib	6	?	?	+	+	+	!
NCT00070499	Dasatinib	6,1	?	?	+	+	+	!
NCT00481247	Dasatinib	21,2	+	?	+	+	+	!
NCT00103844	Dasatinib	1,1	?	?	+	+	+	!
NCT01460693	Dasatinib	27,2	+	?	+	+	+	!
NCT00852566	Dasatinib	0,4	?	?	+	+	?	!

+ Low risk

? Some concerns

– High risk

(B). Arterial occlusive event

Study ID	Experimental	Weight	Randomization process	Deviations from intended interven	Missing outcome data	Measurement of the outcome	Selection of the reported re sult	Overall
NCT01650805	Ponatinib	6,6	+	?	+	+	?	!
NCT00574873	Bosutinib	3,9	+	?	+	+	?	!
NCT02130557	Bosutinib	14,3	+	?	+	?	–	–
NCT00471497	Nilotinib	25,8	+	?	+	+	?	!
NCT00760877	Nilotinib	8,1	?	?	+	+	–	–
NCT01275196	Nilotinib	0,5	?	?	+	?	–	–
NCT00802841	Nilotinib	2,4	?	?	+	+	–	–
NCT00070499	Dasatinib	5,6	?	?	+	?	?	!
NCT00481247	Dasatinib	22,9	+	?	+	+	?	!
NCT00103844	Dasatinib	3,3	?	?	+	+	?	!
NCT00320190	Dasatinib	1,3	?	?	+	+	?	!
NCT01460693	Dasatinib	4,8	+	?	+	?	?	!
NCT00852566	Dasatinib	0,5	?	?	+	+	?	!

+ Low risk
? Some concerns
– High risk

(C). MMR at 12 months

Study ID	Experimental	Weight	Randomization process	Deviations from intended interven	Missing outcome data	Measurement of the outcome	Selection of the reported result	Overall
NCT00574876	Bosutinib	11,8	+	?	+	+	?	!
NCT02130557	Bosutinib	15,4	+	?	+	+	+	!
NCT00471497	Nilotinib	14,2	+	?	+	+	+	!
NCT00760877	Nilotinib	0,7	?	?	+	+	?	!
NCT01275196	Nilotinib	5,3	?	?	+	+	+	!
NCT00802841	Nilotinib	4,6	?	?	+	+	+	!
NCT01400074	Nilotinib	1,5	?	?	+	+	?	!
NCT00070499	Dasatinib	4,2	?	?	+	+	+	!
NCT00481247	Dasatinib	12,1	+	?	+	+	?	!
NCT01460693	Dasatinib	21,6	+	?	+	+	?	!
NCT01593254	Dasatinib	3,1	?	?	+	+	+	!
NCT00852566	Dasatinib	0,6	?	?	+	+	+	!

\+ Low risk
? Some concerns
– High risk

(D). MMR at 24 months

Study ID	Experimental	Weight	Randomization process	Deviations from intended interven	Missing outcome data	Measurement of the outcome	Selection of the reported result	Overall
NCT00574877	Bosutinib	26,1	+	?	+	+	?	!
NCT02130557	Bosutinib	25,5	+	?	+	+	?	!
NCT01275196	Nilotinib	12,2	?	?	+	+	+	!
NCT00802841	Nilotinib	10,3	?	?	+	+	+	!
NCT00481247	Dasatinib	23,9	+	?	+	+	?	!
NCT00852566	Dasatinib	2	?	?	+	+	?	!

\+ Low risk
? Some concerns
– High risk

(E). CCyR at 12 months

Study ID	Experimental	Weight	Randomization process	Deviations from intended interven	Missing outcome data	Measurement of the outcome	Selection of the reported result	Overall
NCT01650806	Ponatinib	0,1	+	?	+	+	+	!
NCT00574878	Bosutinib	14,7	+	?	+	+	+	!
NCT02130557	Bosutinib	11,6	+	?	+	+	?	!
NCT00471497	Nilotinib	14,7	+	?	+	+	+	!
NCT01275196	Nilotinib	7,9	?	?	+	+	?	!
NCT00802841	Nilotinib	7,1	?	?	+	+	+	!
NCT00070499	Dasatinib	2	?	?	+	+	+	!
NCT00481247	Dasatinib	12,6	+	?	+	+	?	!
NCT01460693	Dasatinib	22,8	+	?	+	+	?	!
NCT00852566	Dasatinib	0,1	?	?	+	+	+	!
NCT01511289	Radotinib	6,4	+	?	+	+	?	!

+	Low risk
?	Some concerns
−	High risk

(F). Venous thromboembolism

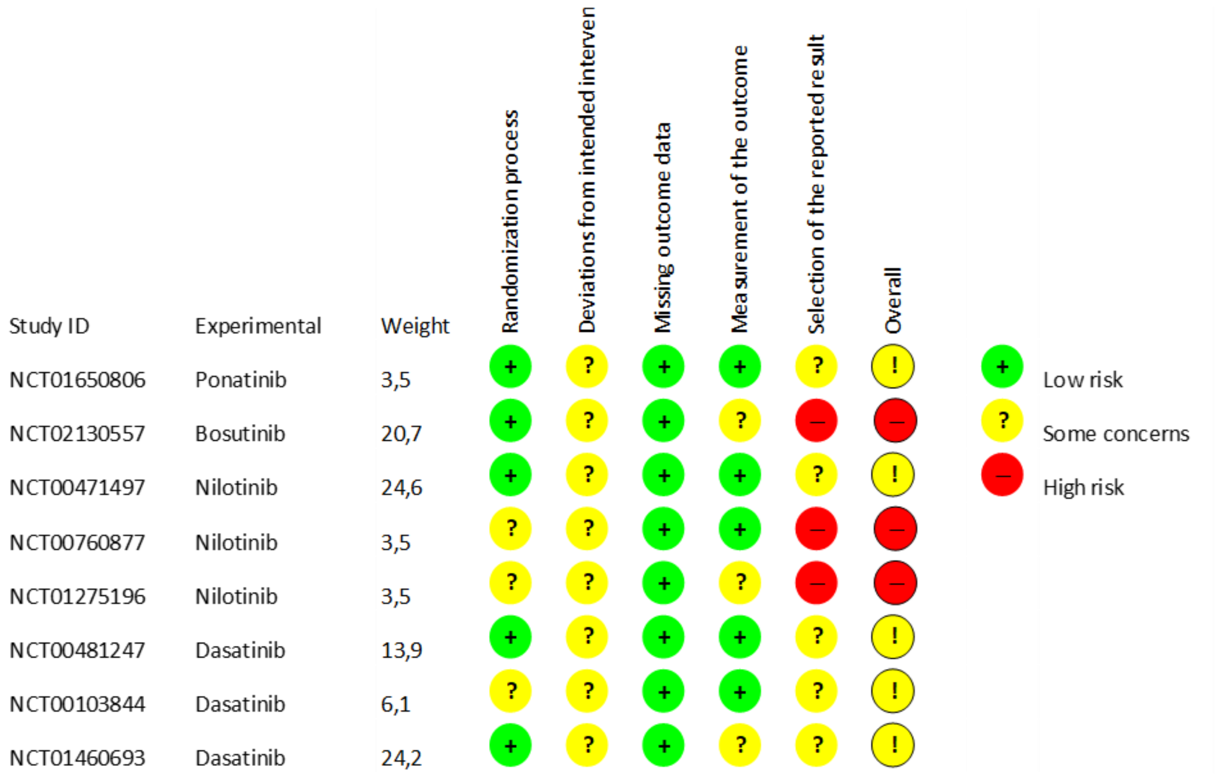

Figure S1. Risk of bias summary. This figure summarizes review authors' judgements about each risk of bias item for each included study. (**A**). Overall survival (**B**). Arterial occlusive event (**C**). MMR at 12 months (**D**). MMR at 24 months (**E**). CCyR at 12 months (**F**). Venous thromboembolism.

(A) Forest plot of AOE stratified by nilotinib dose

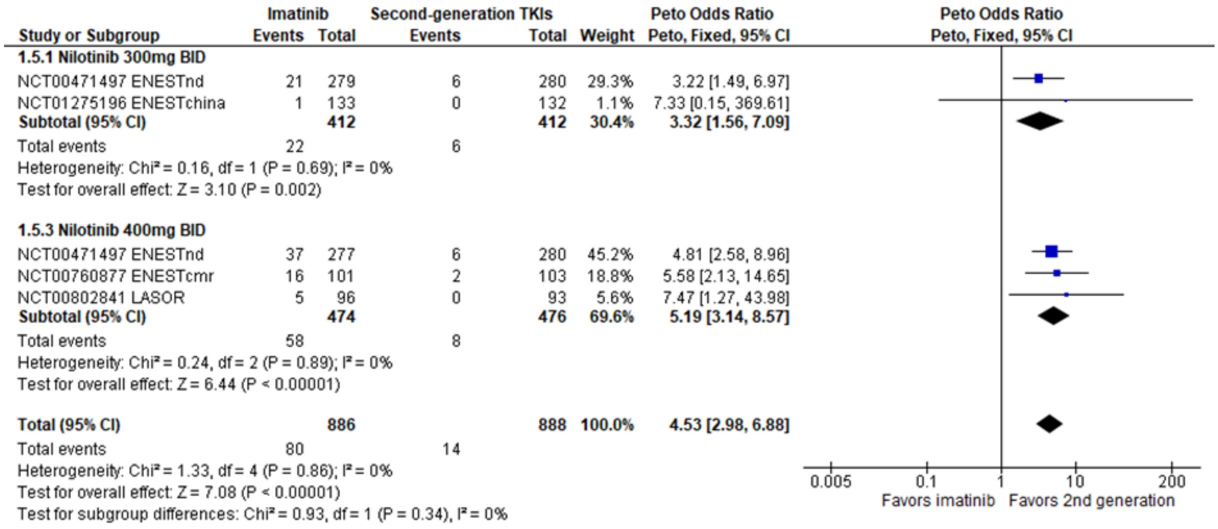

(B) Forest plot of VTE stratified by nilotinib dose

Study or Subgroup	Imatinib Events	Imatinib Total	Second-generation TKIs Events	Second-generation TKIs Total	Weight	Peto Odds Ratio Peto, Fixed, 95% CI
**1.6.1 Nilotinib 300mg BID**						
NCT00471497 ENESTnd	2	279	1	280	27.3%	1.96 [0.20, 18.91]
NCT01275196 ENESTchina	0	133	1	132	9.1%	0.13 [0.00, 6.77]
**Subtotal (95% CI)**		**412**		**412**	**36.5%**	**1.00 [0.14, 7.12]**
Total events	2		2			
Heterogeneity: Chi² = 1.35, df = 1 (P = 0.25); I² = 26%						
Test for overall effect: Z = 0.00 (P = 1.00)						
**1.6.3 Nilotinib 400mg BID**						
NCT00471497 ENESTnd	5	277	1	280	54.4%	3.88 [0.78, 19.37]
NCT00760877 ENESTcmr	0	101	1	103	9.1%	0.14 [0.00, 6.96]
**Subtotal (95% CI)**		**378**		**383**	**63.5%**	**2.40 [0.54, 10.63]**
Total events	5		2			
Heterogeneity: Chi² = 2.38, df = 1 (P = 0.12); I² = 58%						
Test for overall effect: Z = 1.15 (P = 0.25)						
**Total (95% CI)**		**790**		**795**	**100.0%**	**1.74 [0.53, 5.71]**
Total events	7		4			
Heterogeneity: Chi² = 4.21, df = 3 (P = 0.24); I² = 29%						
Test for overall effect: Z = 0.92 (P = 0.36)						
Test for subgroup differences: Chi² = 0.49, df = 1 (P = 0.49), I² = 0%						

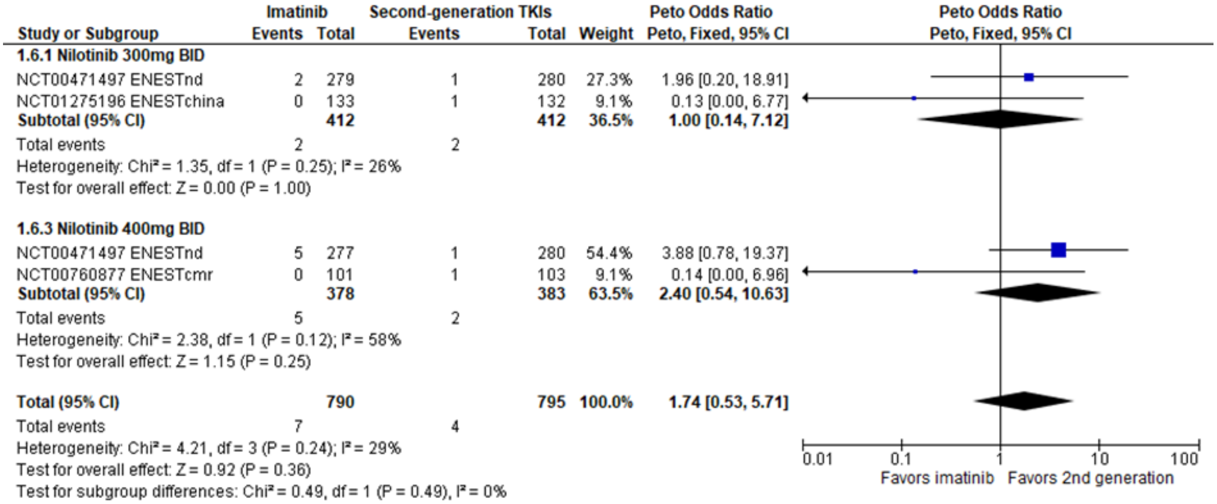

(C) Forest plot of OS stratified by nilotinib dose

Study or Subgroup	Imatinib Events	Imatinib Total	Second-generation TKIs Events	Second-generation TKIs Total	Weight	Odds Ratio M-H, Fixed, 95% CI
**2.2.1 Nilotinib 300mg BID**						
NCT00471497 ENESTnd	264	282	261	283	44.5%	1.24 [0.65, 2.36]
NCT01275196 ENESTchina	132	134	131	133	5.3%	1.01 [0.14, 7.26]
**Subtotal (95% CI)**		**416**		**416**	**49.8%**	**1.21 [0.66, 2.24]**
Total events	396		392			
Heterogeneity: Chi² = 0.04, df = 1 (P = 0.85); I² = 0%						
Test for overall effect: Z = 0.61 (P = 0.54)						
**2.2.3 Nilotinib 400mg BID**						
NCT00471497 ENESTnd	271	281	261	283	24.8%	2.28 [1.06, 4.92]
NCT00760877 ENESTcmr	101	104	100	103	7.8%	1.01 [0.20, 5.12]
NCT00802841 LASOR	89	96	90	95	17.7%	0.71 [0.22, 2.31]
**Subtotal (95% CI)**		**481**		**481**	**50.2%**	**1.53 [0.86, 2.74]**
Total events	461		451			
Heterogeneity: Chi² = 2.94, df = 2 (P = 0.23); I² = 32%						
Test for overall effect: Z = 1.44 (P = 0.15)						
**Total (95% CI)**		**897**		**897**	**100.0%**	**1.37 [0.90, 2.09]**
Total events	857		843			
Heterogeneity: Chi² = 3.24, df = 4 (P = 0.52); I² = 0%						
Test for overall effect: Z = 1.47 (P = 0.14)						
Test for subgroup differences: Chi² = 0.30, df = 1 (P = 0.59), I² = 0%						

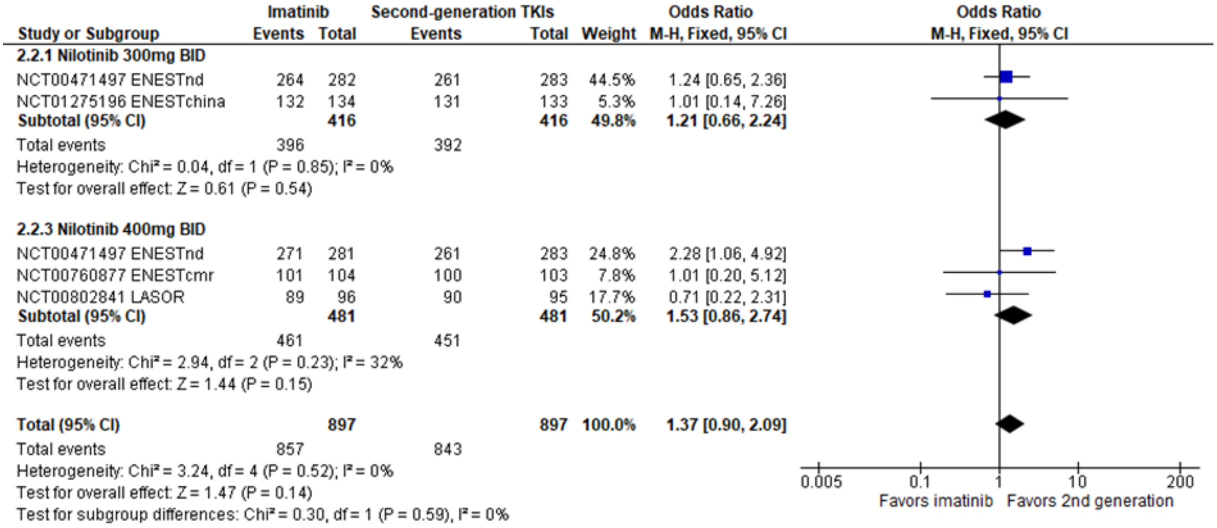

(D) Forest plot of MMR at 12 months stratified by nilotinib dose

Study or Subgroup	Imatinib Events	Imatinib Total	Second-generation TKI Events	Second-generation TKI Total	Weight	Odds Ratio M-H, Fixed, 95% CI
**3.7.1 Nilotinib 300mg**						
NCT00471497 ENESTnd	124	282	63	283	31.7%	2.74 [1.90, 3.95]
NCT01275196 ENESTchina	70	134	37	133	15.9%	2.84 [1.71, 4.72]
**Subtotal (95% CI)**		**416**		**416**	**47.6%**	**2.77 [2.06, 3.73]**
Total events	194		100			
Heterogeneity: Chi² = 0.01, df = 1 (P = 0.91); I² = 0%						
Test for overall effect: Z = 6.73 (P < 0.00001)						
**3.7.3 Nilotinib 400mg**						
NCT00471497 ENESTnd	121	281	63	283	32.1%	2.64 [1.83, 3.81]
NCT00760877 ENESTcmr	20	24	15	28	2.1%	4.33 [1.17, 15.99]
NCT00802841 LASOR	35	96	24	95	13.8%	1.70 [0.91, 3.16]
NCT01400074 RE-NICE	7	22	7	21	4.4%	0.93 [0.26, 3.34]
**Subtotal (95% CI)**		**423**		**427**	**52.4%**	**2.32 [1.72, 3.12]**
Total events	183		109			
Heterogeneity: Chi² = 4.29, df = 3 (P = 0.23); I² = 30%						
Test for overall effect: Z = 5.56 (P < 0.00001)						
**Total (95% CI)**		**839**		**843**	**100.0%**	**2.53 [2.05, 3.13]**
Total events	377		209			
Heterogeneity: Chi² = 5.01, df = 5 (P = 0.41); I² = 0%						
Test for overall effect: Z = 8.69 (P < 0.00001)						
Test for subgroup differences: Chi² = 0.71, df = 1 (P = 0.40), I² = 0%						

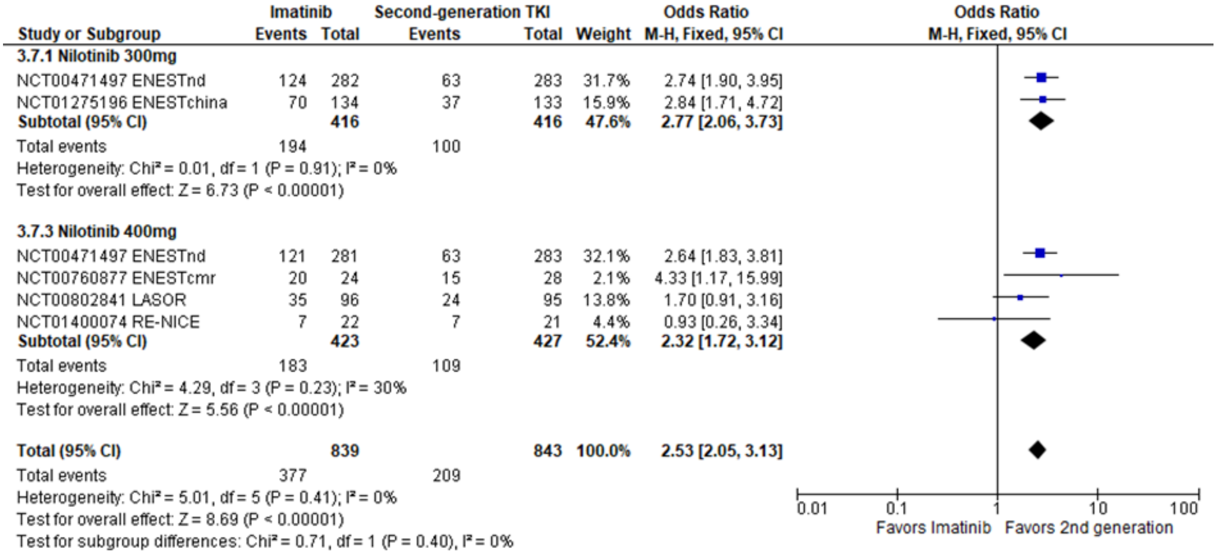

(E) Forest plot of CCyR at 12 months stratified by nilotinib dose

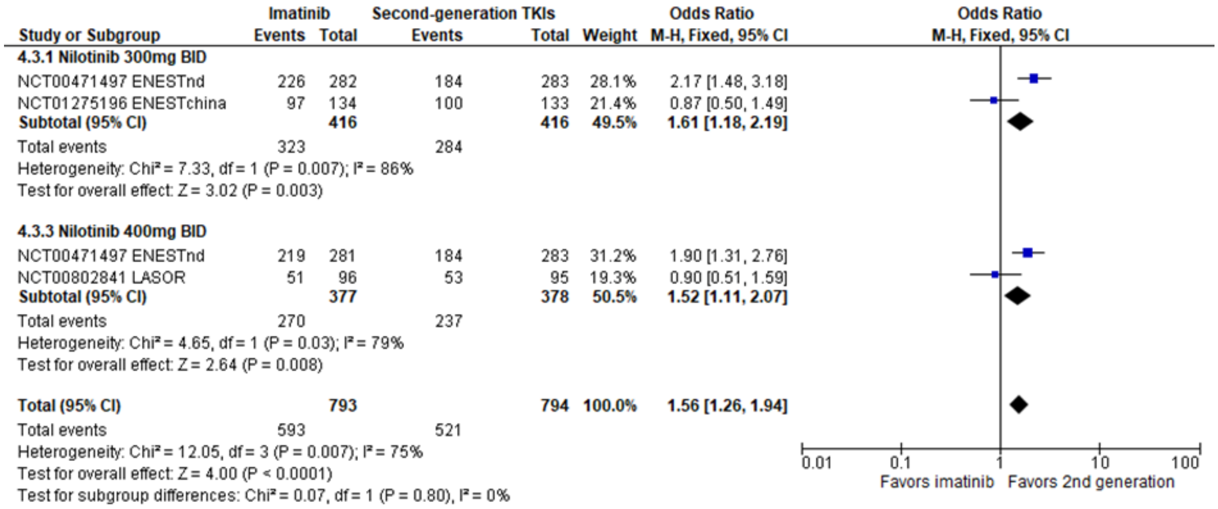

Figure S2. Sub-analyses by TKI dose. (**A**) Forest plot of AOE stratified by nilotinib dose. (**B**) Forest plot of VTE stratified by nilotinib dose. (**C**) Forest plot of OS stratified by nilotinib dose. (**D**) Forest plot of MMR at 12 months stratified by nilotinib dose. (**E**) Forest plot of CCyR at 12 months stratified by nilotinib dose.

(A) Forest plot of MMR at 12 months in treatment-naïve patients.

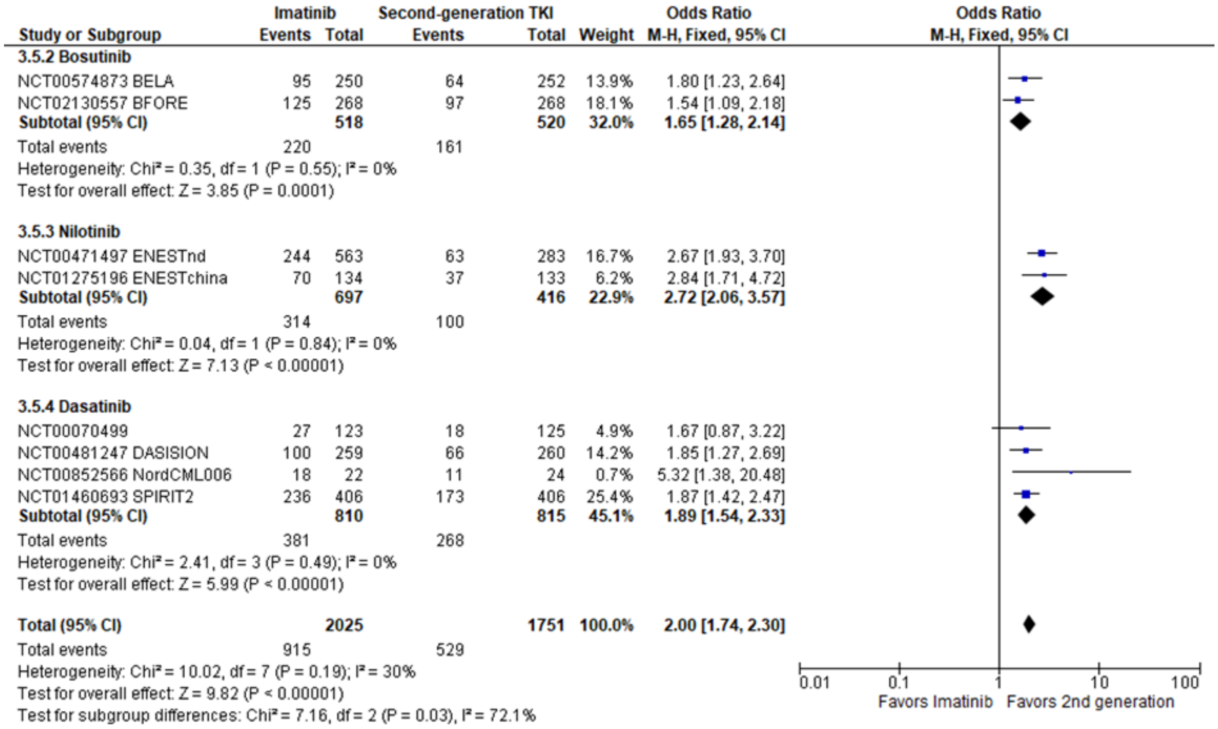

(B) Forest plot of MMR at 24 months in treatment-naïve patients.

Study or Subgroup	Imatinib Events	Imatinib Total	Second-generation TKI Events	Second-generation TKI Total	Weight	Odds Ratio M-H, Fixed, 95% CI
**3.6.2 Bosutinib**						
NCT00574873 BELA	118	250	103	252	29.1%	1.29 [0.91, 1.84]
NCT02130557 BFORE	164	268	136	268	28.4%	1.53 [1.09, 2.16]
**Subtotal (95% CI)**		**518**		**520**	**57.5%**	**1.41 [1.10, 1.80]**
Total events	282		239			
Heterogeneity: Chi² = 0.45, df = 1 (P = 0.50); I² = 0%						
Test for overall effect: Z = 2.74 (P = 0.006)						
**3.6.3 Nilotinib**						
NCT01275196 ENESTchina	83	134	66	133	13.6%	1.65 [1.02, 2.69]
**Subtotal (95% CI)**		**134**		**133**	**13.6%**	**1.65 [1.02, 2.69]**
Total events	83		66			
Heterogeneity: Not applicable						
Test for overall effect: Z = 2.02 (P = 0.04)						
**3.6.4 Dasatinib**						
NCT00481247 DASISION	106	259	84	260	26.6%	1.45 [1.01, 2.08]
NCT00852566 NordCML006	17	22	19	23	2.3%	0.72 [0.16, 3.11]
**Subtotal (95% CI)**		**281**		**283**	**28.9%**	**1.39 [0.98, 1.97]**
Total events	123		103			
Heterogeneity: Chi² = 0.84, df = 1 (P = 0.36); I² = 0%						
Test for overall effect: Z = 1.87 (P = 0.06)						
**Total (95% CI)**		**933**		**936**	**100.0%**	**1.44 [1.19, 1.73]**
Total events	488		408			
Heterogeneity: Chi² = 1.66, df = 4 (P = 0.80); I² = 0%						
Test for overall effect: Z = 3.84 (P = 0.0001)						
Test for subgroup differences: Chi² = 0.37, df = 2 (P = 0.83), I² = 0%						

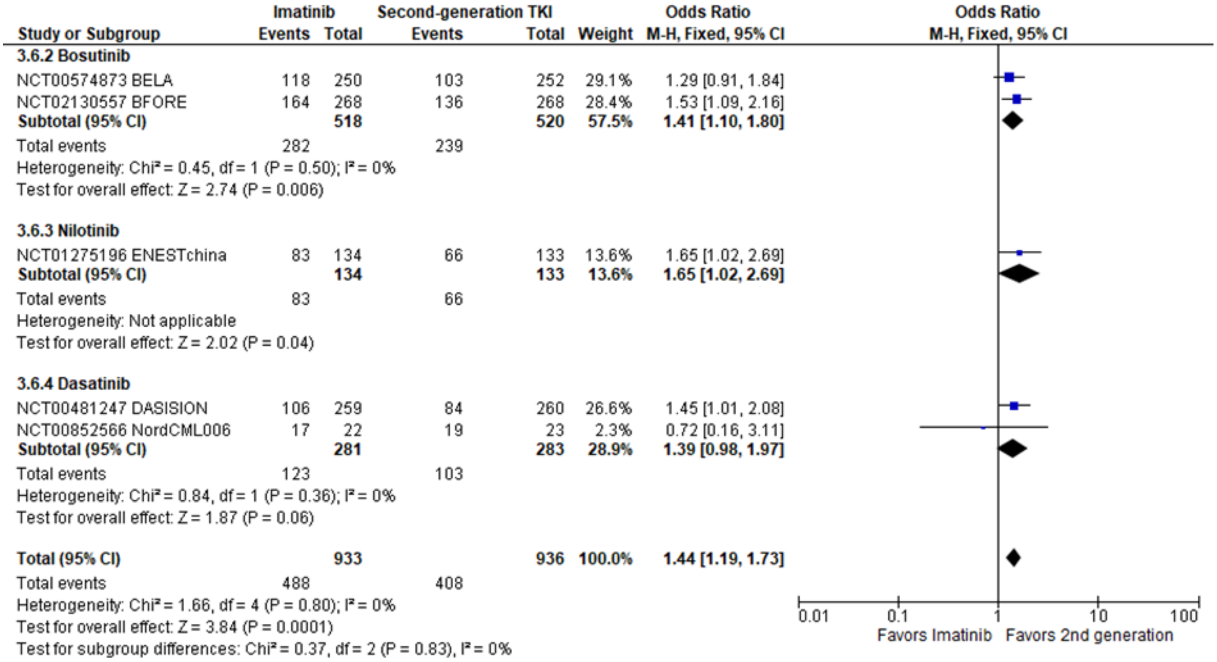

(C) Forest plot of CCyR at 12 months in treatment-naïve patients.

Study or Subgroup	Imatinib Events	Imatinib Total	Second-generation TKIs Events	Second-generation TKIs Total	Weight	Odds Ratio M-H, Fixed, 95% CI
**4.2.2 Bosutinib**						
NCT00574873 BELA	175	250	172	252	17.0%	1.09 [0.74, 1.59]
NCT02130557 BFORE	207	268	178	268	13.4%	1.72 [1.17, 2.51]
**Subtotal (95% CI)**		**518**		**520**	**30.4%**	**1.36 [1.04, 1.78]**
Total events	382		350			
Heterogeneity: Chi² = 2.79, df = 1 (P = 0.10); I² = 64%						
Test for overall effect: Z = 2.27 (P = 0.02)						
**4.2.3 Nilotinib**						
NCT00471497 ENESTnd	445	563	184	283	17.0%	2.03 [1.48, 2.79]
NCT01275196 ENESTchina	97	134	100	133	9.2%	0.87 [0.50, 1.49]
**Subtotal (95% CI)**		**697**		**416**	**26.1%**	**1.62 [1.23, 2.13]**
Total events	542		284			
Heterogeneity: Chi² = 7.00, df = 1 (P = 0.008); I² = 86%						
Test for overall effect: Z = 3.46 (P = 0.0005)						
**4.2.4 Dasatinib**						
NCT00070499	59	70	42	61	2.3%	2.43 [1.05, 5.63]
NCT00481247 DASISION	187	259	159	260	14.6%	1.65 [1.14, 2.39]
NCT00852566 NordCML006	20	20	21	22	0.2%	2.86 [0.11, 74.31]
NCT01460693 SPIRIT2	207	406	163	406	26.4%	1.55 [1.17, 2.05]
**Subtotal (95% CI)**		**755**		**749**	**43.5%**	**1.64 [1.32, 2.03]**
Total events	473		385			
Heterogeneity: Chi² = 1.10, df = 3 (P = 0.78); I² = 0%						
Test for overall effect: Z = 4.51 (P < 0.00001)						
**Total (95% CI)**		**1970**		**1685**	**100.0%**	**1.55 [1.34, 1.79]**
Total events	1397		1019			
Heterogeneity: Chi² = 12.15, df = 7 (P = 0.10); I² = 42%						
Test for overall effect: Z = 6.01 (P < 0.00001)						
Test for subgroup differences: Chi² = 1.23, df = 2 (P = 0.54), I² = 0%						

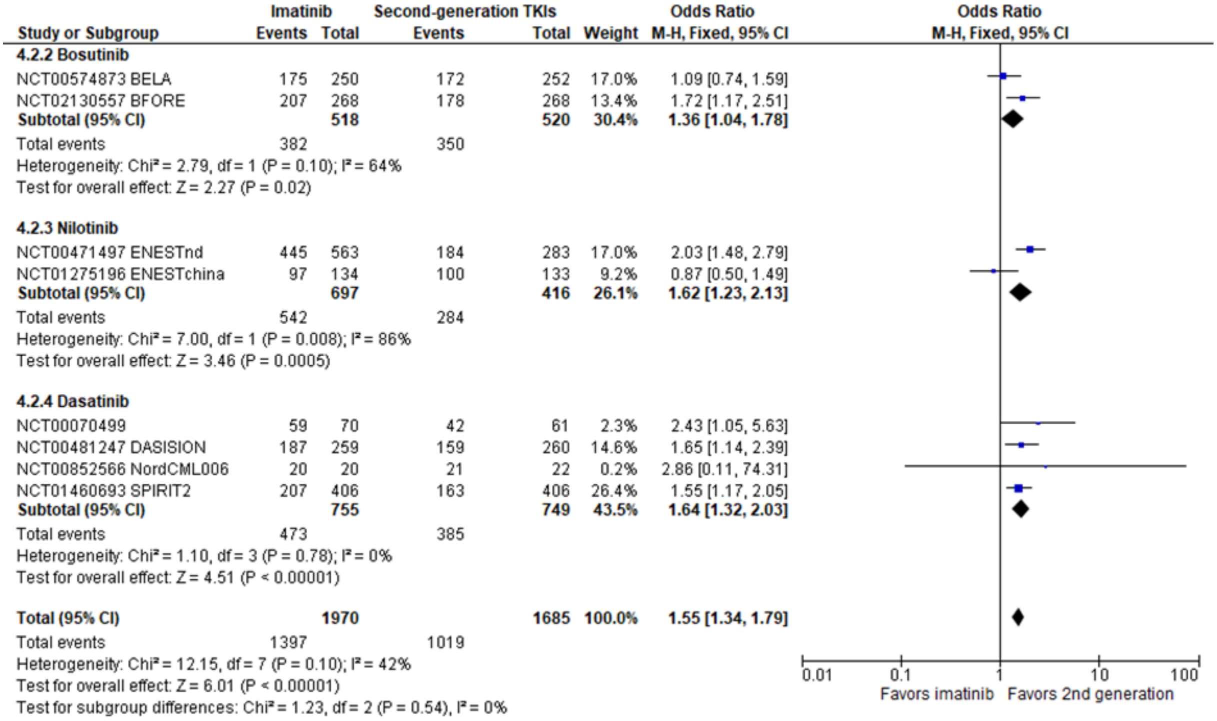

(D) Forest plot of OS in treatment-naïve patients

Study or Subgroup	Imatinib Events	Imatinib Total	Second-generation TKIs Events	Second-generation TKIs Total	Weight	Odds Ratio M-H, Fixed, 95% CI
**2.4.2 Bosutinib**						
NCT00574873 BELA	235	250	237	252	14.6%	0.99 [0.47, 2.07]
NCT02130557 BFORE	265	268	259	268	3.0%	3.07 [0.82, 11.47]
**Subtotal (95% CI)**		**518**		**520**	**17.6%**	**1.34 [0.72, 2.52]**
Total events	500		496			
Heterogeneity: Chi² = 2.16, df = 1 (P = 0.14); I² = 54%						
Test for overall effect: Z = 0.93 (P = 0.35)						
**2.4.3 Nilotinib**						
NCT00471497 ENESTnd	535	563	261	283	17.8%	1.61 [0.90, 2.87]
NCT01275196 ENESTchina	132	134	131	133	2.0%	1.01 [0.14, 7.26]
**Subtotal (95% CI)**		**697**		**416**	**19.8%**	**1.55 [0.89, 2.70]**
Total events	667		392			
Heterogeneity: Chi² = 0.20, df = 1 (P = 0.66); I² = 0%						
Test for overall effect: Z = 1.55 (P = 0.12)						
**2.4.4 Dasatinib**						
NCT00070499	116	123	119	125	6.9%	0.84 [0.27, 2.56]
NCT00481247 DASISION	233	259	234	260	24.2%	1.00 [0.56, 1.77]
NCT00852566 NordCML006	22	22	23	24	0.5%	2.87 [0.11, 74.26]
NCT01460693 SPIRIT2	373	406	370	406	31.0%	1.10 [0.67, 1.80]
**Subtotal (95% CI)**		**810**		**815**	**62.6%**	**1.04 [0.73, 1.49]**
Total events	744		746			
Heterogeneity: Chi² = 0.59, df = 3 (P = 0.90); I² = 0%						
Test for overall effect: Z = 0.24 (P = 0.81)						
**Total (95% CI)**		**2025**		**1751**	**100.0%**	**1.20 [0.92, 1.57]**
Total events	1911		1634			
Heterogeneity: Chi² = 4.44, df = 7 (P = 0.73); I² = 0%						
Test for overall effect: Z = 1.31 (P = 0.19)						
Test for subgroup differences: Chi² = 1.54, df = 2 (P = 0.46), I² = 0%						

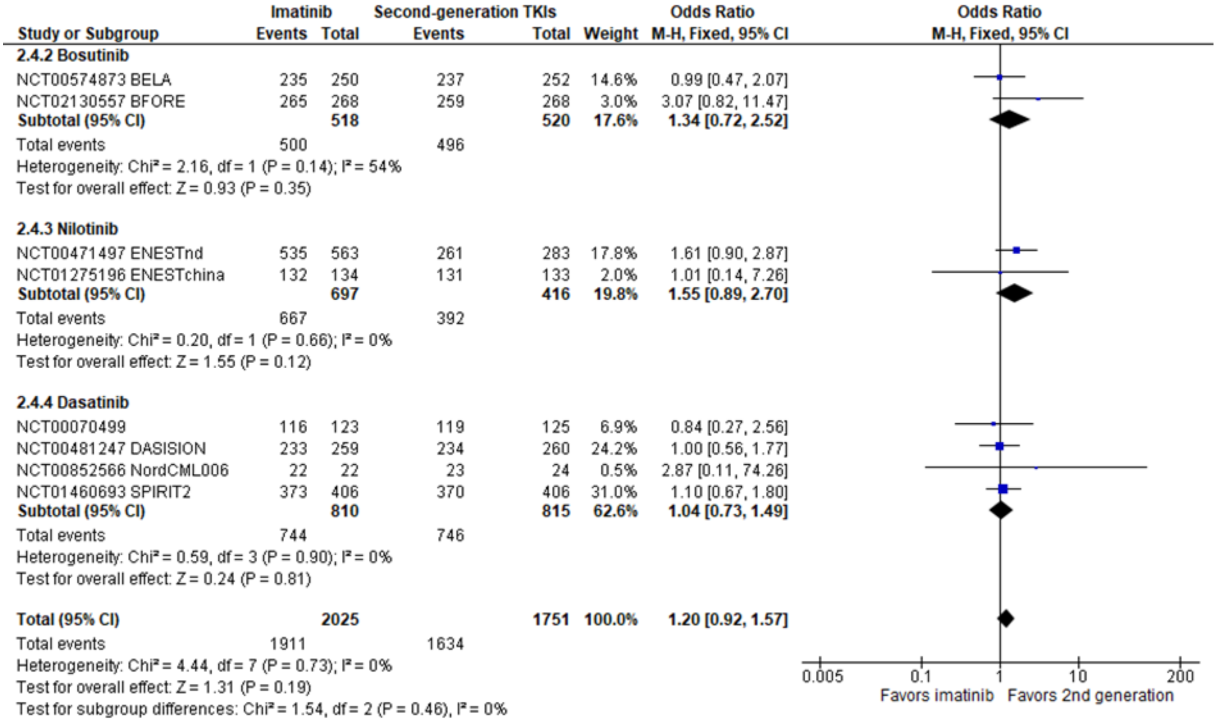

(E) Forest plot of MMR at 12 months in patients with CML treated with second generation TKIs compared with high-dose imatinib.

Study or Subgroup	Imatinib Events	Imatinib Total	Second-generation TKI Events	Second-generation TKI Total	Weight	Odds Ratio M-H, Fixed, 95% CI
**3.8.3 Nilotinib**						
NCT00802841 LASOR	35	96	24	95	49.9%	1.70 [0.91, 3.16]
NCT01400074 RE-NICE	7	22	7	21	15.9%	0.93 [0.26, 3.34]
**Subtotal (95% CI)**		**118**		**116**	**65.8%**	**1.51 [0.87, 2.64]**
Total events	42		31			
Heterogeneity: Chi² = 0.68, df = 1 (P = 0.41); I² = 0%						
Test for overall effect: Z = 1.46 (P = 0.15)						
**3.8.4 Dasatinib**						
NCT01593254 DASCERN	50	174	11	86	34.2%	2.75 [1.35, 5.61]
**Subtotal (95% CI)**		**174**		**86**	**34.2%**	**2.75 [1.35, 5.61]**
Total events	50		11			
Heterogeneity: Not applicable						
Test for overall effect: Z = 2.78 (P = 0.005)						
**Total (95% CI)**		**292**		**202**	**100.0%**	**1.94 [1.25, 2.98]**
Total events	92		42			
Heterogeneity: Chi² = 2.36, df = 2 (P = 0.31); I² = 15%						
Test for overall effect: Z = 2.99 (P = 0.003)						
Test for subgroup differences: Chi² = 1.67, df = 1 (P = 0.20), I² = 40.3%						

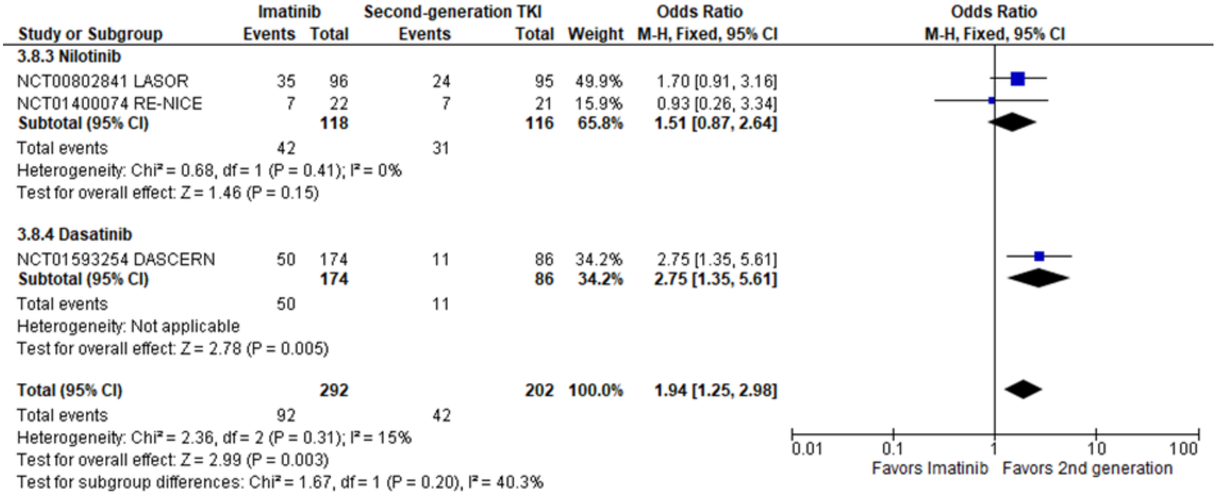

(F) Forest plot of OS in patients with CML treated with second generation TKIs compared with high-dose imatinib.

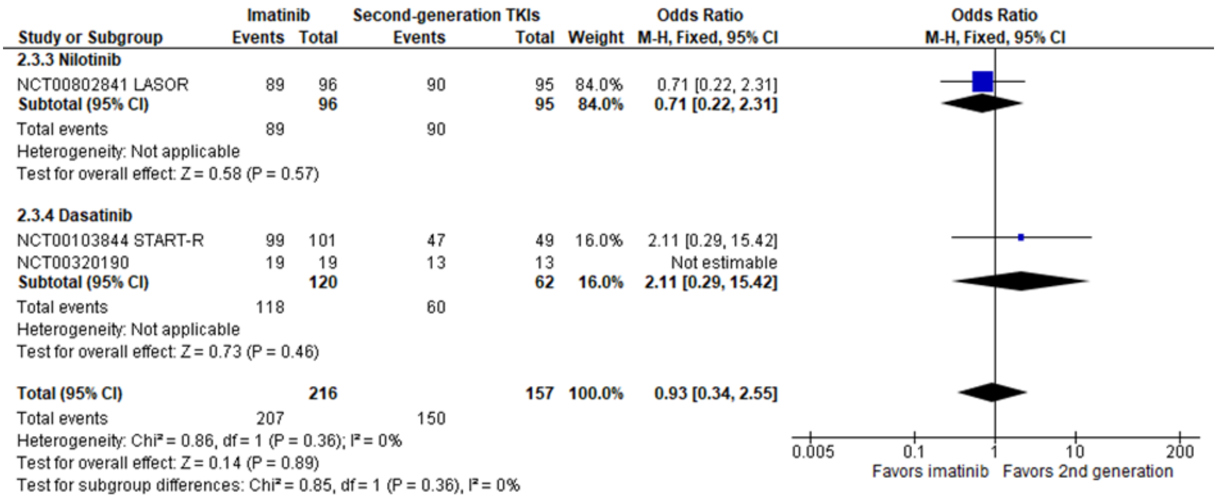

(G) Forest plot of AOE in patients with CML treated with second generation TKIs compared with high-dose imatinib.

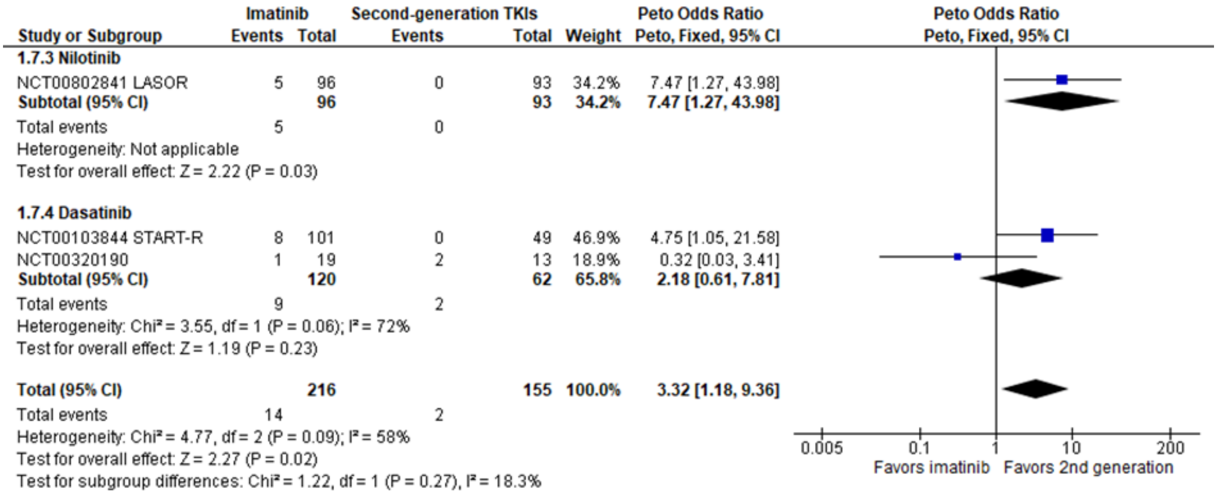

Figure S3. Sensitivity analysis. (**A**) Forest plot of MMR at 12 months in treatment-naïve patients. (**B**) Forest plot of MMR at 24 months in treatment-naïve patients. (**C**) Forest plot of CCyR at 12 months in treatment-naïve patients. (**D**) Forest plot of OS in treatment-naïve patients (**E**) Forest plot of MMR at 12 months in patients with CML treated with second generation TKIs compared with high-dose imatinib. (**F**) Forest plot of OS in patients with CML treated with second generation TKIs compared with high-dose imatinib. (**G**) Forest plot of AOE in patients with CML treated with second generation TKIs compared with high-dose imatinib.

(A) Forest plot of MMR at 12 months.

Study or Subgroup	Imatinib Events	Imatinib Total	Second-generation TKI Events	Second-generation TKI Total	Weight	Odds Ratio M-H, Fixed, 95% CI
**3.1.2 Bosutinib**						
NCT00574873 BELA	95	250	64	252	12.4%	1.80 [1.23, 2.64]
NCT02130557 BFORE	125	268	97	268	16.3%	1.54 [1.09, 2.18]
**Subtotal (95% CI)**		**518**		**520**	**28.7%**	**1.65 [1.28, 2.14]**
Total events	220		161			
Heterogeneity: Chi² = 0.35, df = 1 (P = 0.55); I² = 0%						
Test for overall effect: Z = 3.85 (P = 0.0001)						
**3.1.3 Nilotinib**						
NCT00471497 ENESTnd	244	563	63	283	14.9%	2.67 [1.93, 3.70]
NCT00760877 ENESTcmr	20	24	15	28	0.7%	4.33 [1.17, 15.99]
NCT00802841 LASOR	35	96	24	95	4.8%	1.70 [0.91, 3.16]
NCT01275196 ENESTchina	70	134	37	133	5.6%	2.84 [1.71, 4.72]
NCT01400074 RE-NICE	7	22	7	21	1.5%	0.93 [0.26, 3.34]
**Subtotal (95% CI)**		**839**		**560**	**27.6%**	**2.48 [1.95, 3.16]**
Total events	376		146			
Heterogeneity: Chi² = 4.85, df = 4 (P = 0.30); I² = 18%						
Test for overall effect: Z = 7.40 (P < 0.00001)						
**3.1.4 Dasatinib**						
NCT00070499	27	123	18	125	4.4%	1.67 [0.87, 3.22]
NCT00481247 DASISION	100	259	66	260	12.7%	1.85 [1.27, 2.69]
NCT00852566 NordCML006	18	22	11	24	0.6%	5.32 [1.38, 20.48]
NCT01460693 SPIRIT2	236	406	173	406	22.8%	1.87 [1.42, 2.47]
NCT01593254 DASCERN	50	174	11	86	3.3%	2.75 [1.35, 5.61]
**Subtotal (95% CI)**		**984**		**901**	**43.7%**	**1.96 [1.60, 2.39]**
Total events	431		279			
Heterogeneity: Chi² = 3.40, df = 4 (P = 0.49); I² = 0%						
Test for overall effect: Z = 6.59 (P < 0.00001)						
**Total (95% CI)**		**2341**		**1981**	**100.0%**	**2.01 [1.77, 2.30]**
Total events	1027		586			
Heterogeneity: Chi² = 13.78, df = 11 (P = 0.25); I² = 20%						
Test for overall effect: Z = 10.44 (P < 0.00001)						
Test for subgroup differences: Chi² = 5.25, df = 2 (P = 0.07), I² = 61.9%						

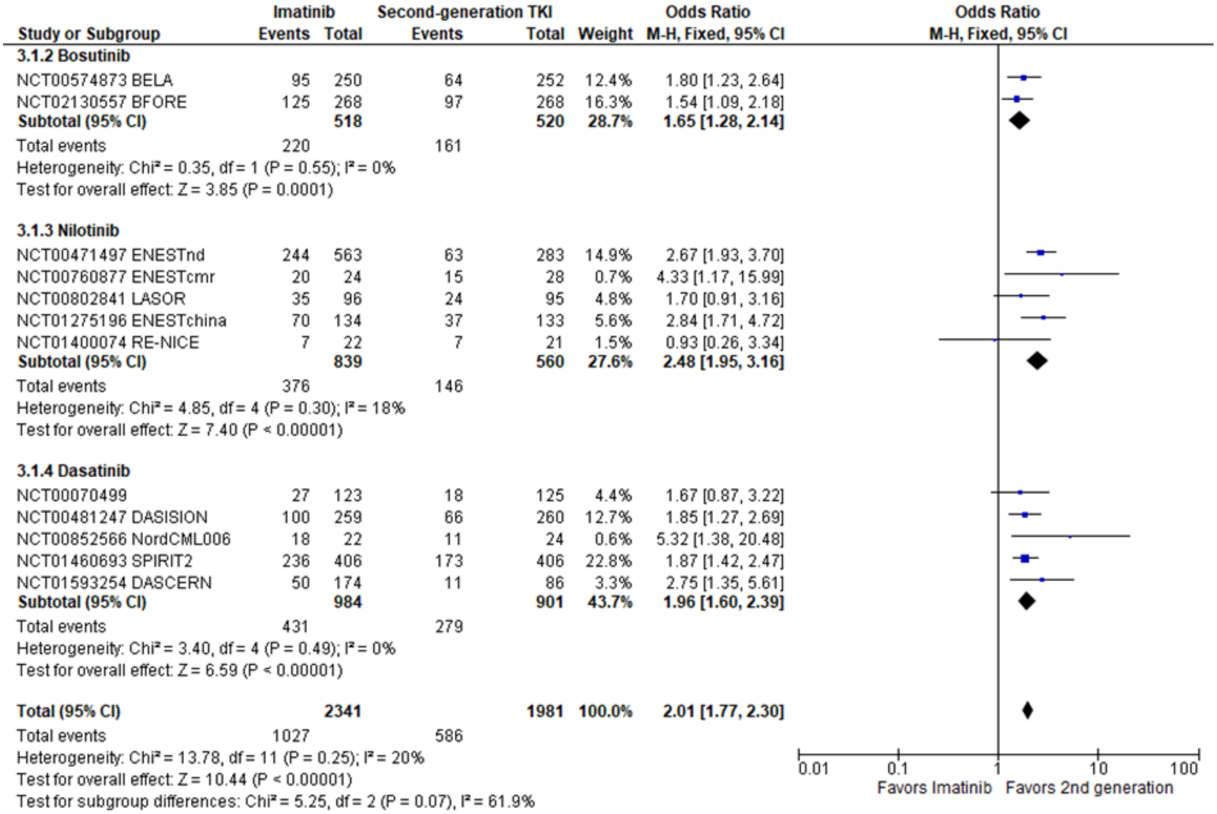

(B) Forest plot of MMR at 24 months.

Study or Subgroup	Imatinib Events	Imatinib Total	Second-generation TKI Events	Second-generation TKI Total	Weight	Odds Ratio M-H, Fixed, 95% CI
**3.2.2 Bosutinib**						
NCT00574873 BELA	118	250	103	252	26.1%	1.29 [0.91, 1.84]
NCT02130557 BFORE	164	268	136	268	25.5%	1.53 [1.09, 2.16]
**Subtotal (95% CI)**		**518**		**520**	**51.6%**	**1.41 [1.10, 1.80]**
Total events	282		239			
Heterogeneity: Chi² = 0.45, df = 1 (P = 0.50); I² = 0%						
Test for overall effect: Z = 2.74 (P = 0.006)						
**3.2.3 Nilotinib**						
NCT00802841 LASOR	36	96	34	95	10.3%	1.08 [0.60, 1.94]
NCT01275196 ENESTchina	83	134	66	133	12.2%	1.65 [1.02, 2.69]
**Subtotal (95% CI)**		**230**		**228**	**22.5%**	**1.39 [0.95, 2.02]**
Total events	119		100			
Heterogeneity: Chi² = 1.21, df = 1 (P = 0.27); I² = 17%						
Test for overall effect: Z = 1.72 (P = 0.09)						
**3.2.4 Dasatinib**						
NCT00481247 DASISION	106	259	84	260	23.9%	1.45 [1.01, 2.08]
NCT00852566 NordCML006	17	22	19	23	2.0%	0.72 [0.16, 3.11]
**Subtotal (95% CI)**		**281**		**283**	**25.9%**	**1.39 [0.98, 1.97]**
Total events	123		103			
Heterogeneity: Chi² = 0.84, df = 1 (P = 0.36); I² = 0%						
Test for overall effect: Z = 1.87 (P = 0.06)						
**Total (95% CI)**		**1029**		**1031**	**100.0%**	**1.40 [1.17, 1.67]**
Total events	524		442			
Heterogeneity: Chi² = 2.50, df = 5 (P = 0.78); I² = 0%						
Test for overall effect: Z = 3.73 (P = 0.0002)						
Test for subgroup differences: Chi² = 0.01, df = 2 (P = 1.00), I² = 0%						

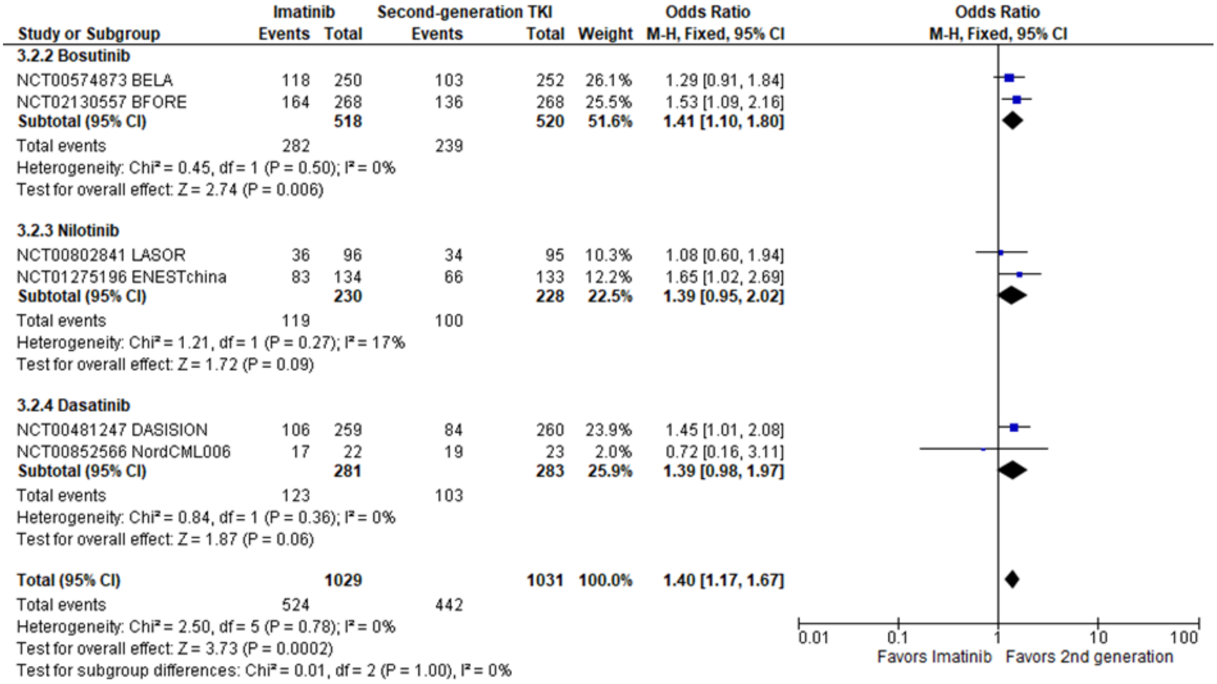

(C) Forest plot of CCyR at 12 months.

Study or Subgroup	Imatinib Events	Imatinib Total	Second-generation TKIs Events	Second-generation TKIs Total	Weight	Odds Ratio M-H, Fixed, 95% CI
**4.1.2 Bosutinib**						
NCT00574873 BELA	175	250	172	252	15.7%	1.09 [0.74, 1.59]
NCT02130557 BFORE	207	268	178	268	12.4%	1.72 [1.17, 2.51]
**Subtotal (95% CI)**		**518**		**520**	**28.1%**	**1.36 [1.04, 1.78]**
Total events	382		350			
Heterogeneity: Chi² = 2.79, df = 1 (P = 0.10); I² = 64%						
Test for overall effect: Z = 2.27 (P = 0.02)						
**4.1.3 Nilotinib**						
NCT00471497 ENESTnd	445	563	184	283	15.7%	2.03 [1.48, 2.79]
NCT00802841 LASOR	51	96	53	95	7.6%	0.90 [0.51, 1.59]
NCT01275196 ENESTchina	97	134	100	133	8.5%	0.87 [0.50, 1.49]
**Subtotal (95% CI)**		**793**		**511**	**31.8%**	**1.45 [1.13, 1.85]**
Total events	593		337			
Heterogeneity: Chi² = 10.45, df = 2 (P = 0.005); I² = 81%						
Test for overall effect: Z = 2.94 (P = 0.003)						
**4.1.4 Dasatinib**						
NCT00070499	59	70	42	61	2.2%	2.43 [1.05, 5.63]
NCT00481247 DASISION	187	259	159	260	13.5%	1.65 [1.14, 2.39]
NCT00852566 NordCML006	20	20	21	22	0.1%	2.86 [0.11, 74.31]
NCT01460693 SPIRIT2	207	406	163	406	24.4%	1.55 [1.17, 2.05]
**Subtotal (95% CI)**		**755**		**749**	**40.2%**	**1.64 [1.32, 2.03]**
Total events	473		385			
Heterogeneity: Chi² = 1.10, df = 3 (P = 0.78); I² = 0%						
Test for overall effect: Z = 4.51 (P < 0.00001)						
**Total (95% CI)**		**2066**		**1780**	**100.0%**	**1.50 [1.31, 1.72]**
Total events	1448		1072			
Heterogeneity: Chi² = 15.49, df = 8 (P = 0.05); I² = 48%						
Test for overall effect: Z = 5.74 (P < 0.00001)						
Test for subgroup differences: Chi² = 1.20, df = 2 (P = 0.55), I² = 0%						

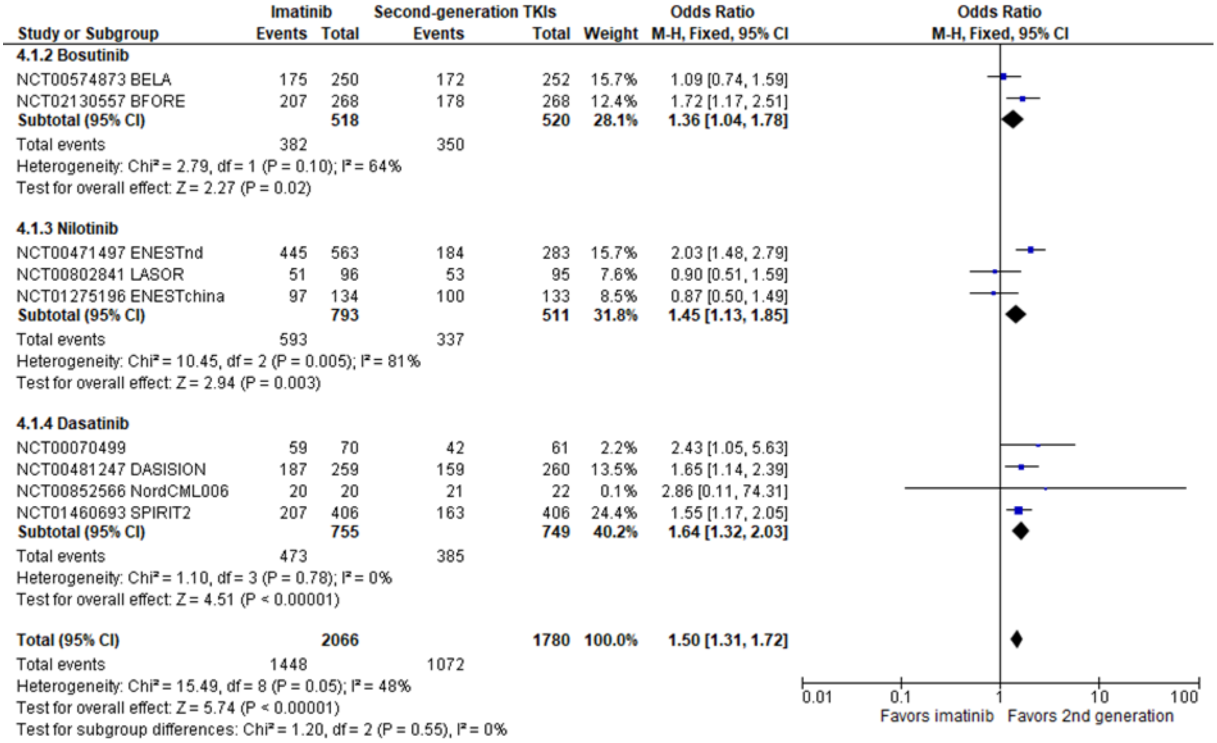

(D) Forest plot of venous thromboembolism.

Study or Subgroup	Imatinib Events	Imatinib Total	Second-generation TKIs Events	Second-generation TKIs Total	Weight	Peto Odds Ratio Peto, Fixed, 95% CI
**1.2.2 Bosutinib**						
NCT00574873 BELA	0	248	0	251		Not estimable
NCT02130557 BFORE	4	268	2	265	21.5%	1.94 [0.39, 9.68]
**Subtotal (95% CI)**		**516**		**516**	**21.5%**	**1.94 [0.39, 9.68]**
Total events	4		2			
Heterogeneity: Not applicable						
Test for overall effect: Z = 0.81 (P = 0.42)						
**1.2.3 Nilotinib**						
NCT00471497 ENESTnd	7	556	1	280	25.5%	2.59 [0.59, 11.30]
NCT00760877 ENESTcmr	0	101	1	103	3.6%	0.14 [0.00, 6.96]
NCT00802841 LASOR	0	96	0	93		Not estimable
NCT01275196 ENESTchina	0	133	1	132	3.6%	0.13 [0.00, 6.77]
**Subtotal (95% CI)**		**886**		**608**	**32.8%**	**1.35 [0.37, 4.97]**
Total events	7		3			
Heterogeneity: Chi² = 3.38, df = 2 (P = 0.18); I² = 41%						
Test for overall effect: Z = 0.45 (P = 0.65)						
**1.2.4 Dasatinib**						
NCT00070499	0	123	0	123		Not estimable
NCT00103844 START-R	2	101	0	49	6.3%	4.46 [0.23, 86.51]
NCT00320190	0	19	0	13		Not estimable
NCT00481247 DASISION	3	258	1	258	14.4%	2.73 [0.38, 19.52]
NCT00852566 NordCML006	0	22	0	24		Not estimable
NCT01460693 SPIRIT2	4	406	3	406	25.1%	1.33 [0.30, 5.90]
**Subtotal (95% CI)**		**929**		**873**	**45.8%**	**1.97 [0.66, 5.93]**
Total events	9		4			
Heterogeneity: Chi² = 0.66, df = 2 (P = 0.72); I² = 0%						
Test for overall effect: Z = 1.21 (P = 0.23)						
**Total (95% CI)**		**2331**		**1997**	**100.0%**	**1.74 [0.82, 3.66]**
Total events	20		9			
Heterogeneity: Chi² = 4.25, df = 6 (P = 0.64); I² = 0%						
Test for overall effect: Z = 1.45 (P = 0.15)						
Test for subgroup differences: Chi² = 0.21, df = 2 (P = 0.90), I² = 0%						

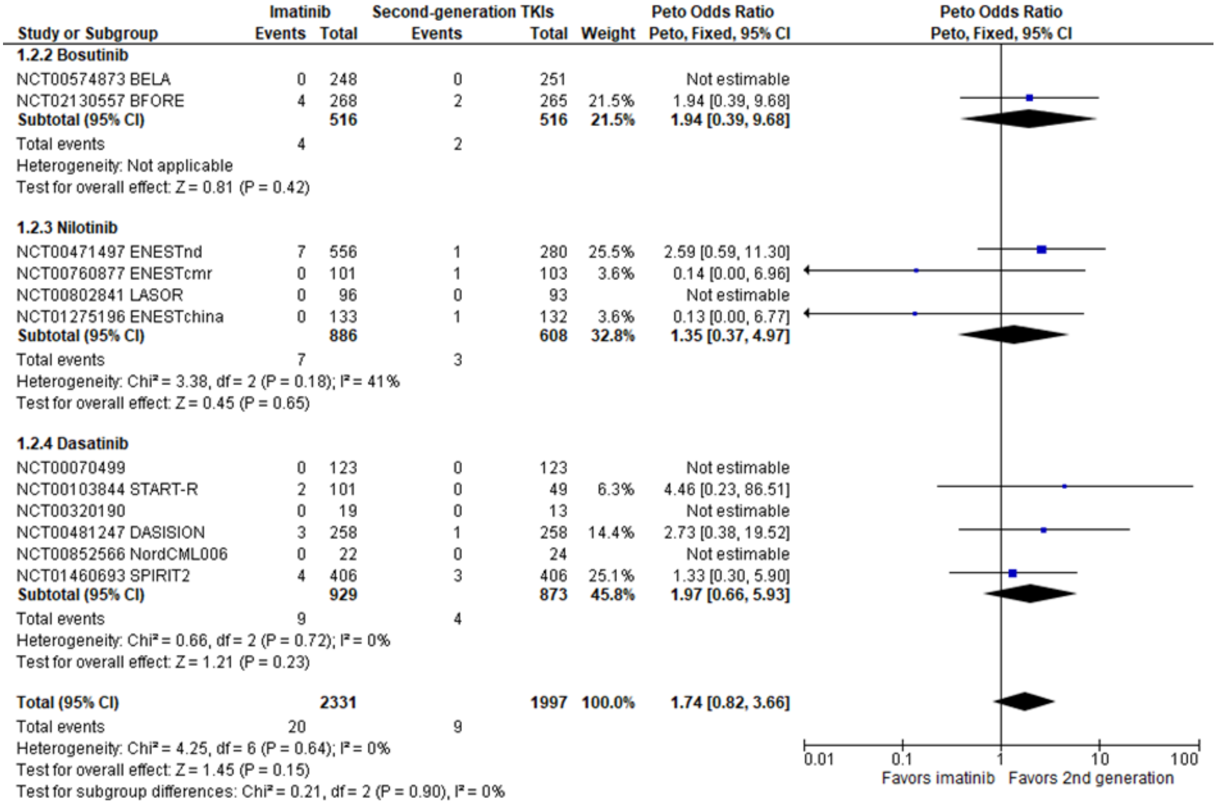

Figure S4. Forest plots of MMR, CCyR and venous thromboembolism in patients with CML treated with second generation TKIs compared with imatinib. (**A**) Forest plot of MMR at 12 months. (**B**) Forest plot of MMR at 24 months. (**C**) Forest plot of CCyR at 12 months. (**D**) Forest plot of venous thromboembolism.

(A) Funnel plot of AOE analysis. Egger's test for a regression intercept gave a *p*-value of 0.489 indicating no evidence of publication bias.

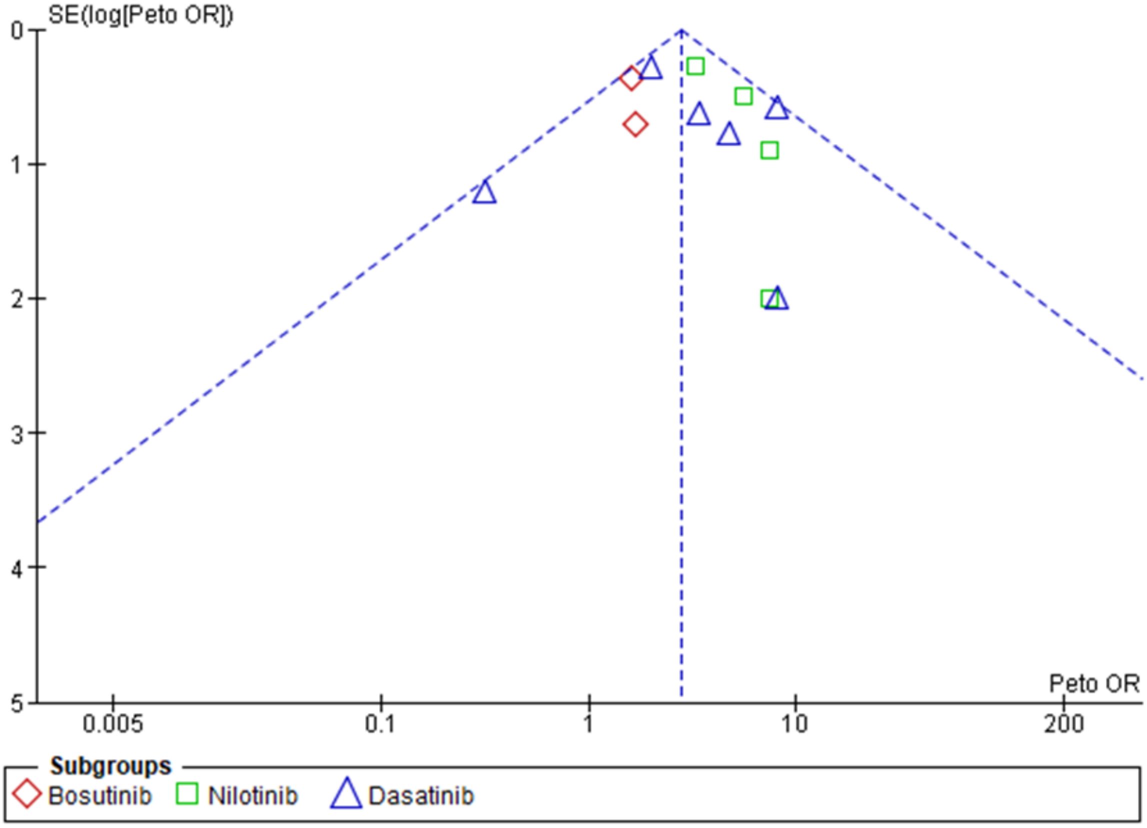

(B) Funnel plot of VTE analysis. Egger's test was not performed for this funnel plot as less than 10 studies was included in the VTE analysis.

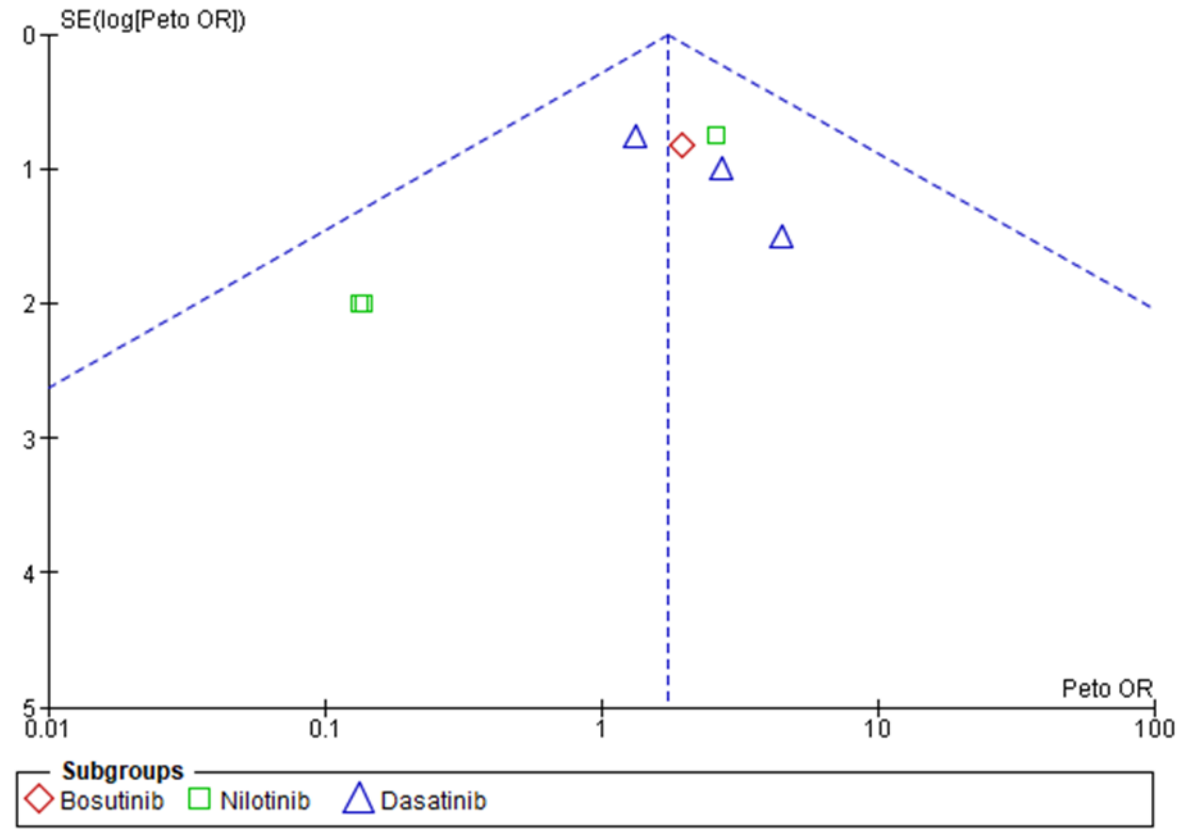

(C) Funnel plot of OS analysis. Egger's test for a regression intercept gave a *p*-value of 0.470 indicating no evidence of publication bias.

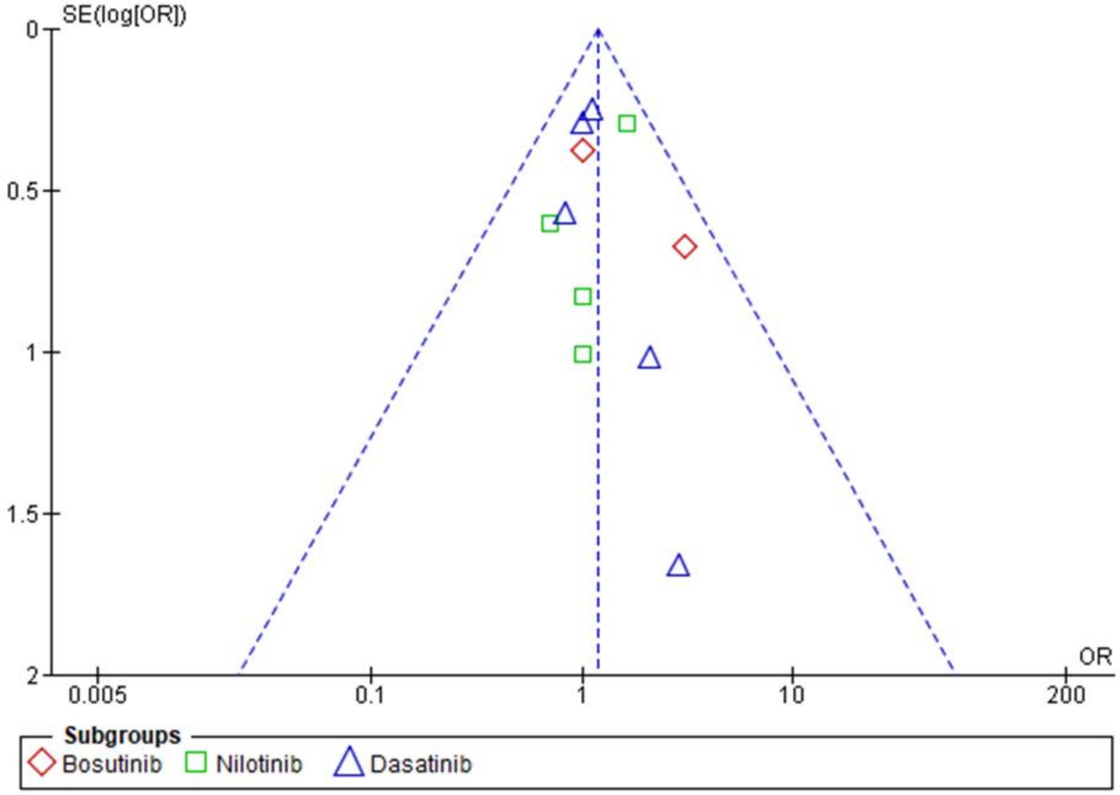

(D) Funnel plot of MMR at 12 months analysis. Egger's test for a regression intercept gave a *p*-value of 0.583 indicating no evidence of publication bias.

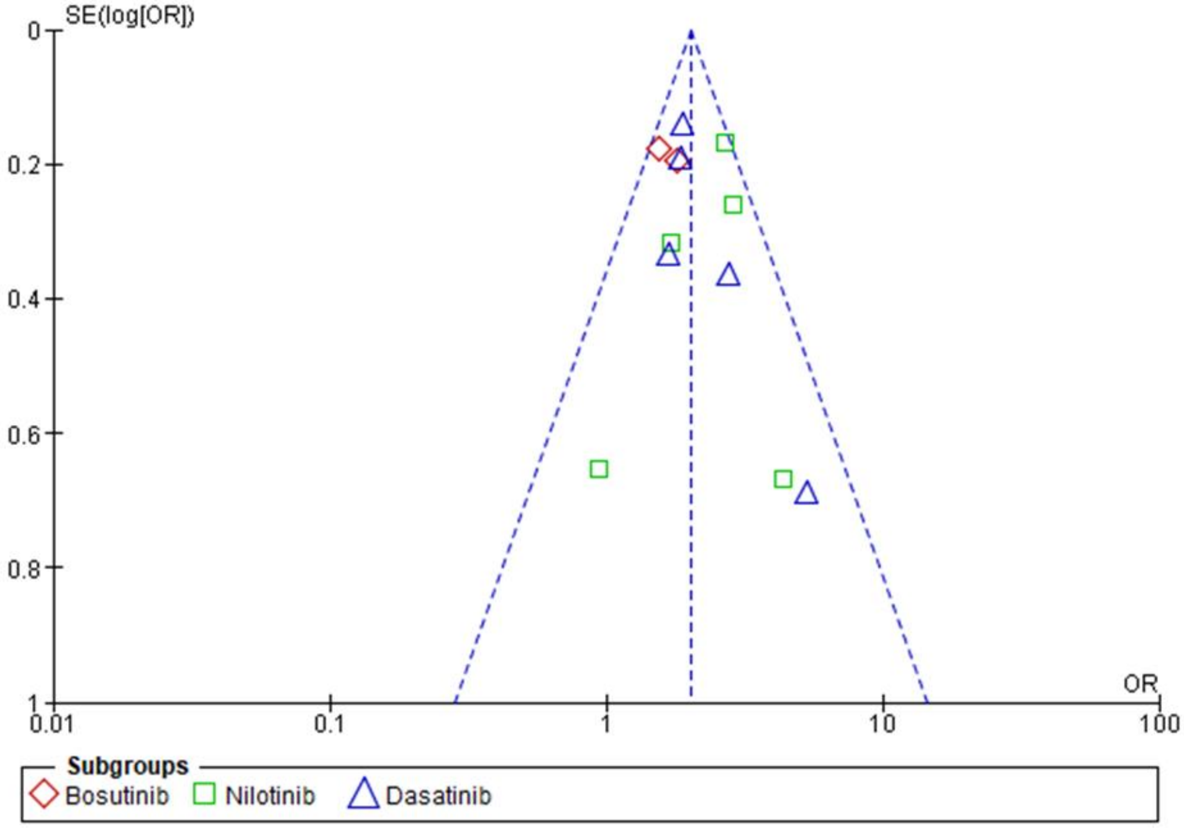

(E) Funnel plot of MMR at 24 months analysis. Egger's test was not performed for this funnel plot as less than 10 studies was included in the MMR at 24 months analysis.

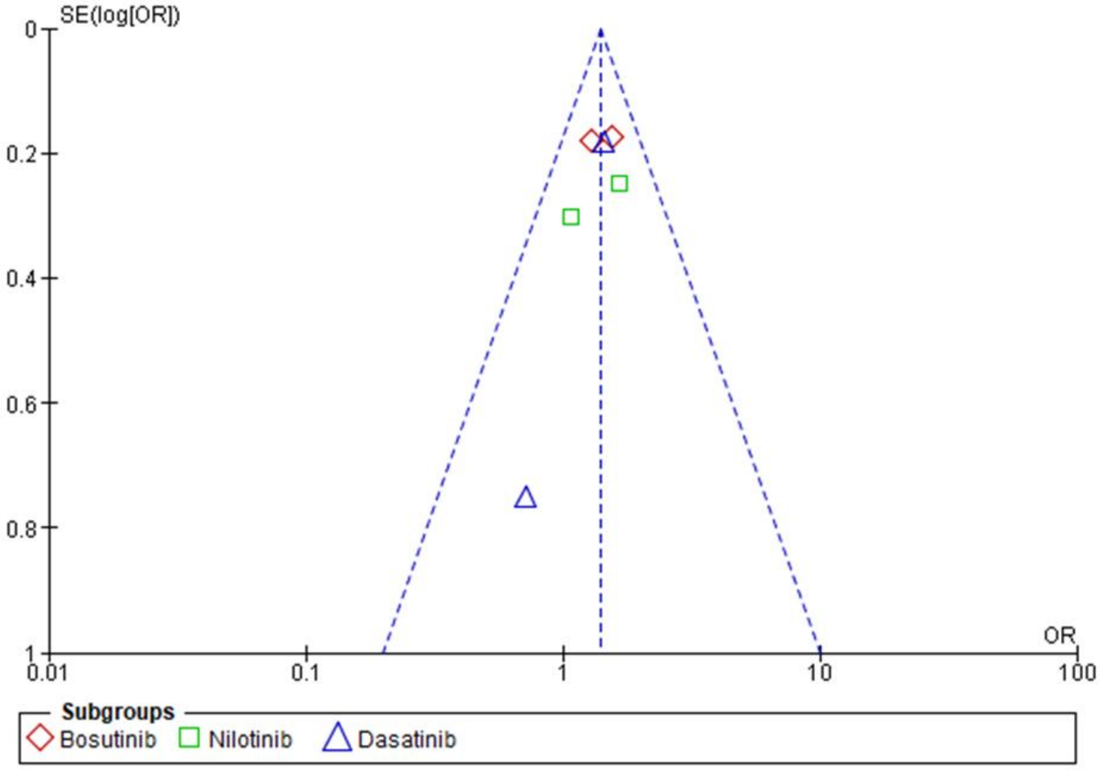

(F) Funnel plot of CCyR at 12 months analysis. Egger's test was not performed for this funnel plot as less than 10 studies was included in the CCyR at 12 months analysis.

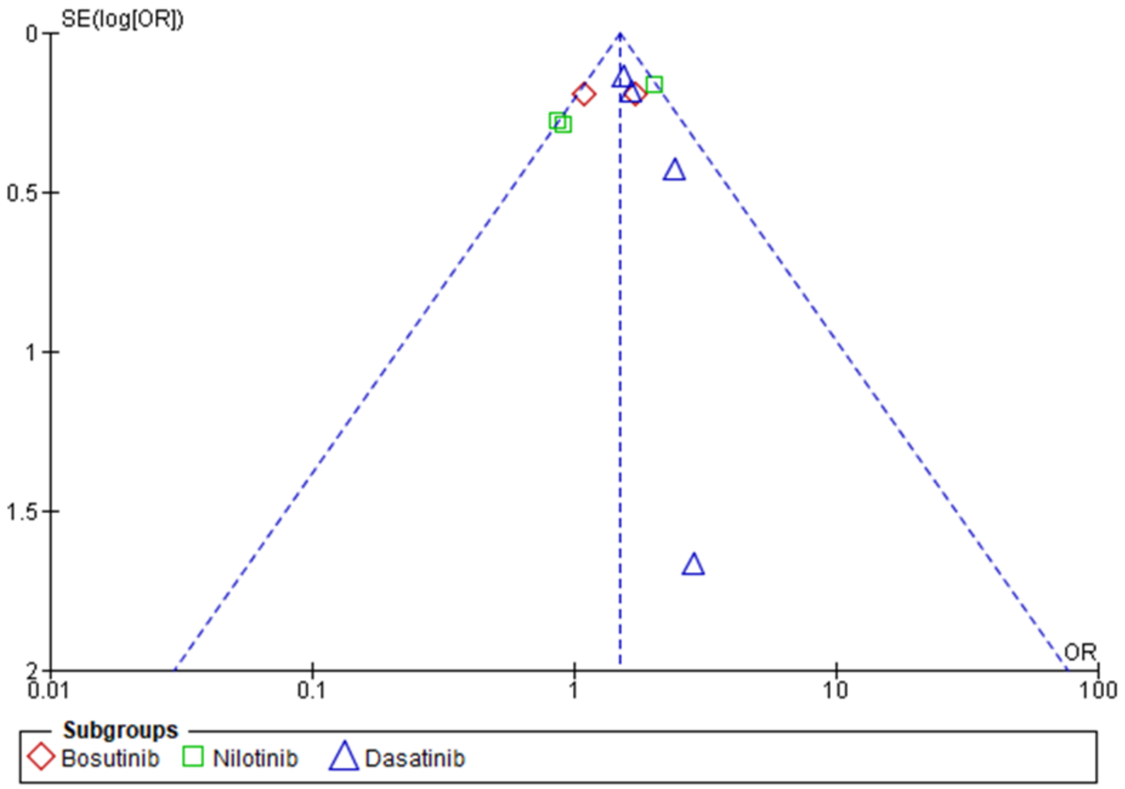

Figure S5. Publication bias assessment. (**A**) Funnel plot of AOE analysis. (**B**) Funnel plot of VTE analysis. (**C**) Funnel plot of OS analysis. (**D**) Funnel plot of MMR at 12 months analysis (**E**) Funnel plot of MMR at 24 months analysis (**F**) Funnel plot of CCyR at 12 months analysis.

Table S1. Main characteristics of the 14 included clinical trials.

NCT Study Name	Study Design	NG-TKI Dosage Frequency	Imatinib Dosage Frequency	Nb of Patients (ITT)	Population	Age (Mean ± SD)	Sex (% Male)	Primary Endpoint	Secondary Endpoint(s)	Key Inclusion Criteria	Key Exclusion Criteria
NCT00574873 BELA	randomized open-label	Bosutinib 500 mg QD	Imatinib 400 mg QD	502	newly diagnosed CP-CML	46.5 ± 14.61	56.6%	CCyR at 12 months	MMR at 12 months	- Cytogenetic diagnosis of CP Ph+ CML diagnosed less than 6 months.<br>- Diagnosis of CML chronic phase confirmed.<br>- Adequate hepatic and renal function.	- Philadelphia negative CML.<br>- Prior anti-leukemia treatment.<br>- Prior stem cell transplant.
NCT02130557 BFORE	randomized open-label	Bosutinib 400 mg QD	Imatinib 400 mg QD	536	newly diagnosed CP-CML	53.0	58.0%	MMR at 12 months	MMR by 18 months<br>CCyR by 12 months<br>OS at 12 months	- Molecular diagnosis of CP CML of ≤ 6 months.<br>- Adequate hepatic, renal and pancreatic function.	- Any prior medical treatment for CML, including TKIs, with the exception of hydroxyurea and/or anagrelide treatment<br>- Any past or current Central Nervous System involvement.<br>- Extramedullary disease only.<br>- Major surgery or radiotherapy within 14 days of randomization.<br>**- History of clinically significant or uncontrolled cardiac disease.**<br>- History of another malignancy within 5 years (exception accepted)
NCT00471497 ENESTnd	randomized open-label	Nilotinib 300 mg BID<br>Nilotinib 400 mg BID	Imatinib 400 mg QD	846	newly diagnosed CP-CML	46.7	58.0%	MMR at 12 months	Durable MMR at 24 months<br>CCyR at 12 months	- CML in CP patients within the first 6 months of diagnosis.<br>- Diagnosis of CML in CP with confirmation of Philadelphia chromosome of (9:22) translocations	- Previously documented T315I mutation<br>- Treatment with a TKI prior to study entry<br>- Any medical treatment for CML with the exception of hydroxyurea and/or anagrelide<br>**- Impaired cardiac function.**<br>**- Severe or uncontrolled medical conditions (i.e., uncontrolled diabetes, active or uncontrolled infection).**<br>- Use of therapeutic coumarin derivatives (i.e., warfarin, acenocoumarol, phenprocoumon)<br>**- Currently receiving treatment with any medications that have the potential to prolong the QT interval.**

NCT00760877 ENESTcmr	randomized open-label	Nilotinib 400 mg BID	Imatinib 400 mg QD Imatinib 600 mg QD	207	CP-CML previously treated with imatinib for at least 2 years	49.1 ± 13.16	65.7%	Rate of best CMR	OS	- Diagnosis of CML associated with BCR-ABL quantifiable by RQ-PCR<br>- Documented CCyR by bone marrow or BCR-ABL < 1% IS in the past 12 months<br>- Persistent disease demonstrated by 2 PCR positive tests 3 months apart both during the past 6 months.<br>- Treatment with imatinib for at least 2 years with 400 mg or 600 mg and a stable dose<br>- No other current or planned anti-leukemia therapies	- Evidence of rising PCR<br>- Treatment with another investigational agent within last 6 months or TKIs other than imatinib<br>- Prior allogeneic stem cell transplantation<br>**- Impaired cardiac function including: inability to monitor the QT interval on electrocardiogram, long QT syndrome or a known family history of long QT syndrome, clinically significant resting brachycardia (< 50 beats per minute), QTc > 450 msec on baseline ECG, other clinically significant uncontrolled heart disease (e.g., unstable angina, congestive heart failure or uncontrolled hypertension), history of or presence of clinically significant ventricular or atrial tachyarrhythmias**<br>- Administration of cytokine therapy (e.g., G-CSF, GM-CSF or SCF) within 4 weeks prior to study entry
NCT01275196 ENESTchina	randomized open-label	Nilotinib 300 mg BID	Imatinib 400 mg QD	267	newly diagnosed CP-CML Chinese patients	40.6 ± 12.82	64.4%	MMR at 12 months	MMR rate at 3, 6, 9, 12, 15, 18, 21, 24 and 36 months<br>OS	- Patients of Chinese ethnicity<br>- Patients with CML-CP (Ph+) within 6 months of diagnosis<br>- No evidence of extramedullary leukemic involvement, with the exception of hepatosplenomegaly<br>- Adequate organ function	- Previously documented T315I mutations.<br>- Treatment with TKIs prior to study entry<br>- Treatment with IFN for more than 3 months.<br>**- Impaired cardiac function including any one of the following: complete left bundle branch block, long QT syndrome or a known family history of long QT syndrome, history of or presence of clinically significant ventricular or atrial tachyarrhythmias, clinically significant resting bradycardia (< 50 beats per minute), QTc > 450 msec, history of clinically documented myocardial infarction within past 12 months, history of unstable angina during the last 12 months, other clinically significant heart disease (e.g., congestive heart failure or uncontrolled hypertension).**<br>**- Severe or uncontrolled medical conditions (i.e., uncontrolled diabetes, active or uncontrolled infection).**<br>- History of significant congenital or acquired bleeding disorder unrelated to cancer.<br>- Major surgery within 4 weeks.<br>- Treatment with other investigational agents within 30 days.<br>- Another primary malignancy except if the other primary malignancy is neither currently clinically significant or requiring active intervention.<br>- Acute or chronic liver, pancreatic or severe renal disease considered unrelated to disease.<br>**- Current intake of any medications that have the potential to prolong the QT interval**

NCT00802841 LASOR	randomized open-label	Nilotinib 400 mg BID	Imatinib 600 mg QD	191	CP-CML with suboptimal response to imatinib standard dose	44.4 ± 14.75	58.6%	CCyR at 6 months	MMR at 12 and 24 months<br>CCyR at 12 and 24 months<br>OS	- Ph+ CML in CP<br>- No evidence of extramedullary leukemia involvement, with the exception of hepatosplenomegaly<br>- Suboptimal response to 400 mg imatinib	- Prior AP or BC CML.<br>- Prior therapy with imatinib in combination with any other CML drug other than Hydroxyurea and/or Anagrelide;<br>- Imatinib therapy started more than 12 months after the date of the original diagnosis.<br>- Unable to tolerate imatinib at 400 mg.<br>- Previous treatment with any other TKI except Glivec and/or CML therapy other than IFN, hydroxyurea, and /or anagrelide; - Myelotoxicity ≥ Grade 2 present at the time of randomization, - Previously documented T315I mutations<br>**- Impaired cardiac function including: long QT syndrome or family history of long QT syndrome, clinically significant resting brachycardia (< 50 bpm), QTcF >450 msec on screening ECG, myocardial infarction ≤ 12 months prior to the first dose of study drug, other clinically significant heart disease (e.g., CHF, uncontrolled hypertension, unstable angina, significant ventricular or atrial tachyarrhythmias)**<br>**- Currently receiving treatment with any medications that have the potential to prolong the QT interval**<br>- History of another primary malignancy that is currently clinically significant or currently requires active intervention;<br>- Any other clinically significant medical or surgical condition which, according to investigators' discretion, should preclude participation;<br>- Use of investigational agent within 28 days prior to enrollment;

NCT01400074 RE-NICE	randomized open-label	Nilotinib 400 mg BID	Imatinib 400 mg BID	43	CP-CML with suboptimal response to imatinib at a minimum dose of 400 mg daily	40.1	74.4%	Cumulative rate of MMR at 12 months	Safety analyses	- Diagnosis of Ph+ CML in CP<br>- Patients with suboptimal molecular response	- Late CP who started imatinib more than 6 months after diagnosis<br>- Prior AP or BP CML<br>- Previously documented T315I mutations.<br>- Intolerance to imatinib 400 mg daily.<br>- Patients treated with imatinib more than 400 mg daily<br>- Achieved prior MMR or CCyR on imatinib and lost response to entering the study.<br>- Previous treatment with interferon or any other TKI except imatinib (allow hydroxyurea or anagrelide)<br>**- Impaired cardiac function**<br>**- Treatment with inhibitors of CYP3A4 or medications well documented to prolong the QT interval are contraindicated**<br>- Any other malignancy that is clinically significant or requires active intervention.<br>**- Severe or uncontrolled medical conditions**<br>- History of significant congenital or acquired bleeding disorder unrelated to cancer.<br>- Previous radiotherapy to ≥ 25% of the bone marrow.<br>- Major surgery within 4 weeks or who have not recovered from prior surgery.<br>- Treatment with other investigational agents within 30 days.
NCT00070499	randomized open-label	Dasatinib 100 mg QD	Imatinib 400 mg QD	248	newly diagnosed CP-CML	48.5	59.8%	MMR rate at 12 months	2-year OS Toxicity	- CML in CP<br>- Registration on this study within 180 days after the date of first being diagnosed with CML.<br>- **With an electrocardiogram within 42 days, and without any of the following cardiac symptoms: uncontrolled angina, congestive heart failure or myocardial infarction within 6 months, diagnosed or suspected congenital long QT syndrome, history of clinically significant ventricular arrhythmias, prolonged corrected QT interval or uncontrolled hypertension**	- Prior treatment for CML with the exception of hydroxyurea and/or anagrelide<br>- Any prior chemotherapy regimen for peripheral blood stem cell mobilization;<br>- Major surgery within 28 days before registration, or without having fully recovered from any other prior major surgery<br>- Other prior malignancy (exception are allowed)<br>- History of significant bleeding disorder unrelated to cancer, including congenital bleeding disorders and acquired bleeding disorder within one year

NCT00481247 DASISION	randomized open-label	Dasatinib 100 mg QD	Imatinib 400 mg QD	519	newly diagnosed CP-CML	46.7 ± 14.2	59.2%	Best confirmed CCyR within 12 months	Participants remaining in cCCyR at 2, 3, 4 and 5 years<br>MMR at any time<br>OS	- CP Philadelphia Chromosome-positive CML	- **Pleural Effusion**<br>- **Uncontrolled cardiovascular disease**<br>- Significant bleeding disorder unrelated to CML<br>- Prior treatment with interferon/imatinib/dasatinib/anti-CML systemic treatments except anagrelide/hydroxyurea
NCT00103844 START-R	randomized open-label	Dasatinib 70 mg BID	Imatinib 400 mg BID	150	CP-CML resistant to imatinib at 400–600 mg daily	51 ± 13.6	50.0%	MCyR at 12 weeks	MMR<br>CCyR after crossover<br>AEs, SAEs, deaths and hematologic toxicities	- Subjects with CP Ph+ CML.<br>- Subjects have not been treated with imatinib at a dose >600 mg/day.<br>- Subjects developed resistance to disease while receiving an imatinib dose 400–600 mg/day.<br>- Able to tolerate imatinib at the highest dose the subject had received in the past.<br>- Adequate renal and hepatic function.	- Prior treatment with imatinib at a dose >600 mg/day.<br>- Subjects who have previously identified specific BCR-ABL mutations.<br>- Previous diagnosis of AP or BC CML.<br>- Intolerance to imatinib at any dose.<br>- Subjects who are eligible and willing to undergo transplantation during the screening period.<br>- Serious uncontrolled medical disorder or active infection.<br>- **Uncontrolled or significant cardiovascular disease.**<br>**- Uncontrolled hypertension.**<br>- Evidence of organ dysfunction.<br>- Use of imatinib within 7 days.<br>- Use of interferon or cytarabine within 14 days.<br>- Use of a targeted small molecule anticancer agent within 14 days.<br>- **Subjects taking certain medications that are accepted to have a risk of causing Torsades de Pointes.**<br>- Subjects taking medications that irreversibly inhibit platelet function or anticoagulants.<br>- Prior therapy with BMS-354825.
NCT00320190	randomized open-label	Dasatinib 100 mg QD	Imatinib 400 mg BID	32	CP-CML with suboptimal response after imatinib 400 mg daily	48.6 ± 14.85	71.9%	MMR rate at 12 months	Death, AEs, treatment-related AEs, SAEs, treatment-related SAEs and AEs leading to discontinuation<br>On-study AEs of special interest<br>CCyR at 6	- CP Ph+ CML demonstrating only a suboptimal response	- Previous diagnosis of AP or BC CML<br>**- Uncontrolled or significant cardiovascular disease**<br>- History of significant bleeding disorder unrelated to CML<br>- Concurrent malignancies<br>- Intolerance of imatinib 400 mg<br>- Prior treatment with imatinib at a dose higher than 400 mg<br>- Prior stem cell transplantation and/or high-dose chemotherapy for CML

									and 12 months		
NCT01460693 SPIRIT2	randomized open-label	Dasatinib 100 mg QD	Imatinib 400 mg QD	812	newly diagnosed CP-CML	54.4	55.6%	5-year EFS	MMR	- Enrolled within 3 months of initial diagnosis of CML-CP<br>- Cytogenetic confirmation of the Philadelphia chromosome or variants of (9;22) translocations<br>- No evidence of extramedullary leukaemic involvement, with the exception of the hepatosplenomegaly.	- Patients with Ph-negative, BCR-ABL-positive disease.<br>- Any prior treatment for CML with: any TKI; busulphan; interferon-alpha; homoharringtonine; cytosine arabinoside; any other investigational agents (hydroxycarbamide and anagrelide are the only drugs permitted).<br>- Patients who received prior chemotherapy.<br>- Patient who have had any form of prior haemopoietic stem cell transplant, either autograft or allograft.<br>- Patients with International normalized ratio (INR) or partial thromboplastin time (PTT) > 1.5 x IULN, with the exception of patients on treatment with oral anticoagulants.<br>- **Patients with uncontrolled medical disease such as diabetes mellitus, thyroid dysfunction, neuropsychiatric disorders, infection, angina, or Grade 3/4 cardiac problems as defined by the New York Heart Association Criteria.**<br>- Patients who have undergone major surgery within 4 weeks, or who have not recovered from prior major surgery.<br>- Patients with a history of another malignancy either currently or within the past five years (exception).
NCT01593254 DASCERN	randomized open-label	Dasatinib 100 mg QD	Imatinib 400 mg or more QD	260	CP-CML without optimal response to imatinib 400 mg	37.0	78.0%	MMR at 12 months of CML treatment	OS<br>Safety profile<br>Cyotgenetic response over time	- CP-CML Ph+ patients with complete hematologic response (CHR) but without one log BCR-ABL reduction (BCR-ABL level >10% IS) 3 months of imatinib 400 mg treatment.<br>- Currently tolerating imatinib 400 mg QD.<br>- Adequate renal function<br>- Adequate hepatic function	- Previous diagnosis of AP or BC<br>- Subjects with clonal evolution in Ph+ cells observed in ≥2 metaphases at baseline bone marrow cytogenetic test, unless the same abnormalities were present at diagnosis.<br>- Subjects with less than CHR after 3 months of imatinib treatment or lost CHR after initial achievement<br>- Documented T315I/A, F317L, or V299L mutations<br>- A serious uncontrolled medical disorder or active infection that would impair the ability of the subject to receive protocol therapy

NCT00852566 NordCML006	randomized open-label	Dasatinib 100 mg QD	Imatinib 400 mg QD	46	newly diagnosed CP-CML	55.6	47.8%	Ph-positive cells in stem cell compartments at 6 months	Molecular and cytogenetic responses at 3, 6, 12 and 18 months	- CML in CP<br>- No evidence of extramedullary leukemia apart from hepatosplenomegaly<br>- Ph+ or variants must be demonstrated by BM cytogenetics, FISH or PCR.<br>- Previously untreated CML in CP, with the exception of hydroxyurea or anagrelide<br>- Enrolled in this study within 90 days after the date of first being diagnosed with CML<br>- Adequate hepatic function<br>- Adequate renal function	- A serious uncontrolled medical disorder or active infection that would impair the ability of the subject to receive protocol therapy.<br>**- Known pleural effusion at baseline.**<br>**- Uncontrolled or significant cardiovascular disease**<br>- History of significant bleeding disorder unrelated to CML.<br>- Prior chemotherapy for peripheral stem cell mobilization.<br>- Prior or concurrent malignancy.<br>- Any prior treatment with interferon, dasatinib or imatinib<br>- Any other prior systemic treatments, with anti-CML activity [except for anagrelide, or hydroxyurea (HU)].<br>**- Current uptake of drugs that are generally accepted to have a risk of causing Torsades de Pointes**.

Abbreviations: AE: adverse event; AP: acute phase; BP: blast phase; CCyR: complete cytogenetic response; CML: chronic myeloid leukemia; CP: chronic phase; ITT: intention-to-treat; MMR: major molecular response; OS: overall survival; Ph+: Philadelphia positive; SAE: serious adverse event; SD: standard deviation; TKI: tyrosine kinase inhibitors.

Table S2. Detailed statistics in Figure S3. (The bold is a summary of the other rows.).

(A). Table Detailed statistics for MMR at 12 months.

BCR-ABL TKI № of Participants (Studies)	Odds Ratio (95% CI)	Anticipated Absolute Effects (95% CI)		
		Risk with Imatinib	Risk with 2nd Generation TKI	Risk Difference with 2nd Generation TKI
All NG-TKIs<br>№ of Participants: 4322<br>(12 Studies)	**OR 2.01<br>(1.77 to 2.30)**	**29.6%**	**45.8%<br>(42.6 to 49.1)**	**+16.2%<br>(+13.1 to +19.6)**
Bosutinib<br>№ of Participants: 1038<br>(2 Studies)	OR 1.65<br>(1.28 to 2.14)	31.0%	42.5%<br>(36.5 to 49)	+11.6%<br>(+5.5 to +18)
Nilotinib<br>№ of Participants: 1399<br>(5 Studies)	OR 2.48<br>(1.95 to 3.16)	26.1%	46.7%<br>(40.7 to 52.7)	+20.6%<br>(+14.7 to +26.6)
Dasatinib<br>№ of Participants: 1885<br>(5 Studies)	OR 1.96<br>(1.60 to 2.39)	31.0%	46.8%<br>(41.8 to 51.7)	15.8% more<br>(+10.8 to +20.8)

(B). Detailed statistics for MMR at 24 months.

BCR-ABL TKI № of Participants (Studies)	Odds Ratio (95% CI)	Anticipated Absolute Effects (95% CI)		
		Risk with Imatinib	Risk with 2nd Generation TKI	Risk Difference with 2nd Generation TKI
All NG-TKIs<br>№ of Participants: 2060<br>(6 Studies)	**OR 1.40<br>(1.17 to 1.67)**	**42.9%**	**51.2%<br>(46.8 to 55.6)**	**+8.4%<br>(+3.9 to +12.7)**
Bosutinib<br>№ of Participants: 1038<br>(2 Studies)	OR 1.41<br>(1.10 to 1.80)	46.0%	54.5%<br>(48.3 to 60.5)	+8.6%<br>(+2.4 to +14.5)
Nilotinib<br>№ of Participants: 458<br>(2 Studies)	OR 1.39<br>(0.95 to 2.02)	43.9%	52.1%<br>(42.6 to 61.2)	+8.2%<br>(−1.3 to +17.4)
Dasatinib<br>№ of Participants: 564<br>(2 Studies)	OR 1.39<br>(0.98 to 1.97)	36.4%	44.3%<br>(35.9 to 53)	+7.9%<br>(−0.5 to +16.6)

(C). Detailed statistics for CCyR at 12 months.

BCR-ABL TKI № of Participants (Studies)	Odds Ratio (95% CI)	Anticipated Absolute Effects (95% CI)		
		Risk with Imatinib	Risk with 2nd Generation TKI	Risk Difference with 2nd Generation TKI
All NG-TKIs<br>№ of Participants: 3846<br>(9 Studies)	**OR 1.50<br>(1.31 to 1.72)**	**60.2%**	**69.4%<br>(66.5 to 72.3)**	**+9.2%<br>(+6.3 to +12)**
Bosutinib<br>№ of Participants: 1038<br>(2 Studies)	OR 1.36<br>(1.04 to 1.78)	67.3%	73.7%<br>(68.2 to 78.6)	+6.4%<br>(+0.9 to +11.3)
Nilotinib<br>№ of Participants: 1304<br>(3 Studies)	OR 1.45<br>(1.13 to 1.85)	65.9%	73.7%<br>(68.6 to 78.2)	+7.8%<br>(+2.7 to +12.2)
Dasatinib<br>№ of Participants: 1504<br>(4 Studies)	OR 1.64<br>(1.32 to 2.03)	51.4%	63.4%<br>(58.3 to 68.2)	+12.0%<br>(+6.9 to +16.8)

(D). Detailed statistics of venous thromboembolism.

BCR-ABL TKI<br>№ of Participants<br>(Studies)	Odds Ratio<br>(95% CI)	Anticipated Absolute Effects (95% CI)		
		Risk with Imatinib	Risk with 2nd Generation TKI	Risk Difference with 2nd Generation TKI
All NG-TKIs<br>№ of Participants: 4328<br>(12 Studies)	**OR 1.74<br>(0.82 to 3.66)**	**0.5%**	**0.8%<br>(0.4 to 1.6)**	**+0.3%<br>(−0.1 to +1.2)**
Bosutinib<br>№ of Participants: 1032<br>(2 Studies)	OR 1.94<br>(0.39 to 9.68)	0.4%	0.7%<br>(0.2 to 3.6)	+0.4%<br>(−0.2 to +3.2)
Nilotinib<br>№ of Participants: 1494<br>(4 Studies)	OR 1.35<br>(0.37 to 4.97)	0.5%	0.7%<br>(0.2 to 2.4)	+0.2%<br>(−0.3 to +1.9)
Dasatinib<br>№ of Participants: 1802<br>(6 Studies)	OR 1.97<br>(0.66 to 5.93)	0.5%	0.9%<br>(0.3 to 2.7)	+0.4%<br>(−0.2 to +2.2)

Table S3. PRISMA checklist.

Section/Topic	#	Checklist Item	Reported on page #
		**TITLE**	
Title	1	Identify the report as a systematic review, meta-analysis, or both.	1
		**ABSTRACT**	
Structured summary	2	Provide a structured summary including, as applicable: background; objectives; data sources; study eligibility criteria, participants, and interventions; study appraisal and synthesis methods; results; limitations; conclusions and implications of key findings; systematic review registration number.	1
		**INTRODUCTION**	
Rationale	3	Describe the rationale for the review in the context of what is already known.	1-2
Objectives	4	Provide an explicit statement of questions being addressed with reference to participants, interventions, comparisons, outcomes, and study design (PICOS).	2
		**METHODS**	
Protocol and registration	5	Indicate if a review protocol exists, if and where it can be accessed (e.g., Web address), and, if available, provide registration information including registration number.	9
Eligibility criteria	6	Specify study characteristics (e.g., PICOS, length of follow-up) and report characteristics (e.g., years considered, language, publication status) used as criteria for eligibility, giving rationale.	9
Information sources	7	Describe all information sources (e.g., databases with dates of coverage, contact with study authors to identify additional studies) in the search and date last searched.	9

Search	8	Present full electronic search strategy for at least one database, including any limits used, such that it could be repeated.	Method S1
Study selection	9	State the process for selecting studies (i.e., screening, eligibility, included in systematic review, and, if applicable, included in the meta-analysis).	9
Data collection process	10	Describe method of data extraction from reports (e.g., piloted forms, independently, in duplicate) and any processes for obtaining and confirming data from investigators.	9
Data items	11	List and define all variables for which data were sought (e.g., PICOS, funding sources) and any assumptions and simplifications made.	9
Risk of bias in individual studies	12	Describe methods used for assessing risk of bias of individual studies (including specification of whether this was done at the study or outcome level), and how this information is to be used in any data synthesis.	10
Summary measures	13	State the principal summary measures (e.g., risk ratio, difference in means).	10
Synthesis of results	14	Describe the methods of handling data and combining results of studies, if done, including measures of consistency (e.g., I2) for each meta-analysis.	10
Risk of bias across studies	15	Specify any assessment of risk of bias that may affect the cumulative evidence (e.g., publication bias, selective reporting within studies).	10
Additional analyses	16	Describe methods of additional analyses (e.g., sensitivity or subgroup analyses, meta-regression), if done, indicating which were pre-specified.	10
		**RESULTS**	
Study selection	17	Give numbers of studies screened, assessed for eligibility, and included in the review, with reasons for exclusions at each stage, ideally with a flow diagram.	2-3 + Figure 1
Study characteristics	18	For each study, present characteristics for which data were extracted (e.g., study size, PICOS, follow-up period) and provide the citations.	Table S1
Risk of bias within studies	19	Present data on risk of bias of each study and, if available, any outcome level assessment (see item 12).	6 + Figure S1
Results of individual studies	20	For all outcomes considered (benefits or harms), present, for each study: (a) simple summary data for each intervention group (b) effect estimates and confidence intervals, ideally with a forest plot.	3-6 + Figures 2-3 + Figure S3
Synthesis of results	21	Present results of each meta-analysis done, including confidence intervals and measures of consistency.	3-6
Risk of bias across studies	22	Present results of any assessment of risk of bias across studies (see Item 15).	6 + Figure S5
Additional analysis	23	Give results of additional analyses, if done (e.g., sensitivity or subgroup analyses, meta-regression [see Item 16]).	5-6 + Figure S4
		**DISCUSSION**	
Summary of evidence	24	Summarize the main findings including the strength of evidence for each main outcome; consider their relevance to key groups (e.g., healthcare providers, users, and policy makers).	6-8
Limitations	25	Discuss limitations at study and outcome level (e.g., risk of bias), and at review-level (e.g., incomplete retrieval of identified research, reporting bias).	8-9
Conclusions	26	Provide a general interpretation of the results in the context of other evidence, and implications for future research.	6-8
		**FUNDING**	
Funding	27	Describe sources of funding for the systematic review and other support (e.g., supply of data); role of funders for the systematic review.	11

© 2020 by the authors. Licensee MDPI, Basel, Switzerland. This article is an open access article distributed under the terms and conditions of the Creative Commons Attribution (CC BY) license (http://creativecommons.org/licenses/by/4.0/).
